# Supplementary figures and images for: Stromal fibroblasts induce metastatic tumor cell clusters via epithelial–mesenchymal plasticity
Source: Life Sci Alliance. 2019 Jul 22;2(4):e201900425. doi: 10.26508/lsa.201900425 (PMC6653778; doi:10.26508/lsa.201900425)

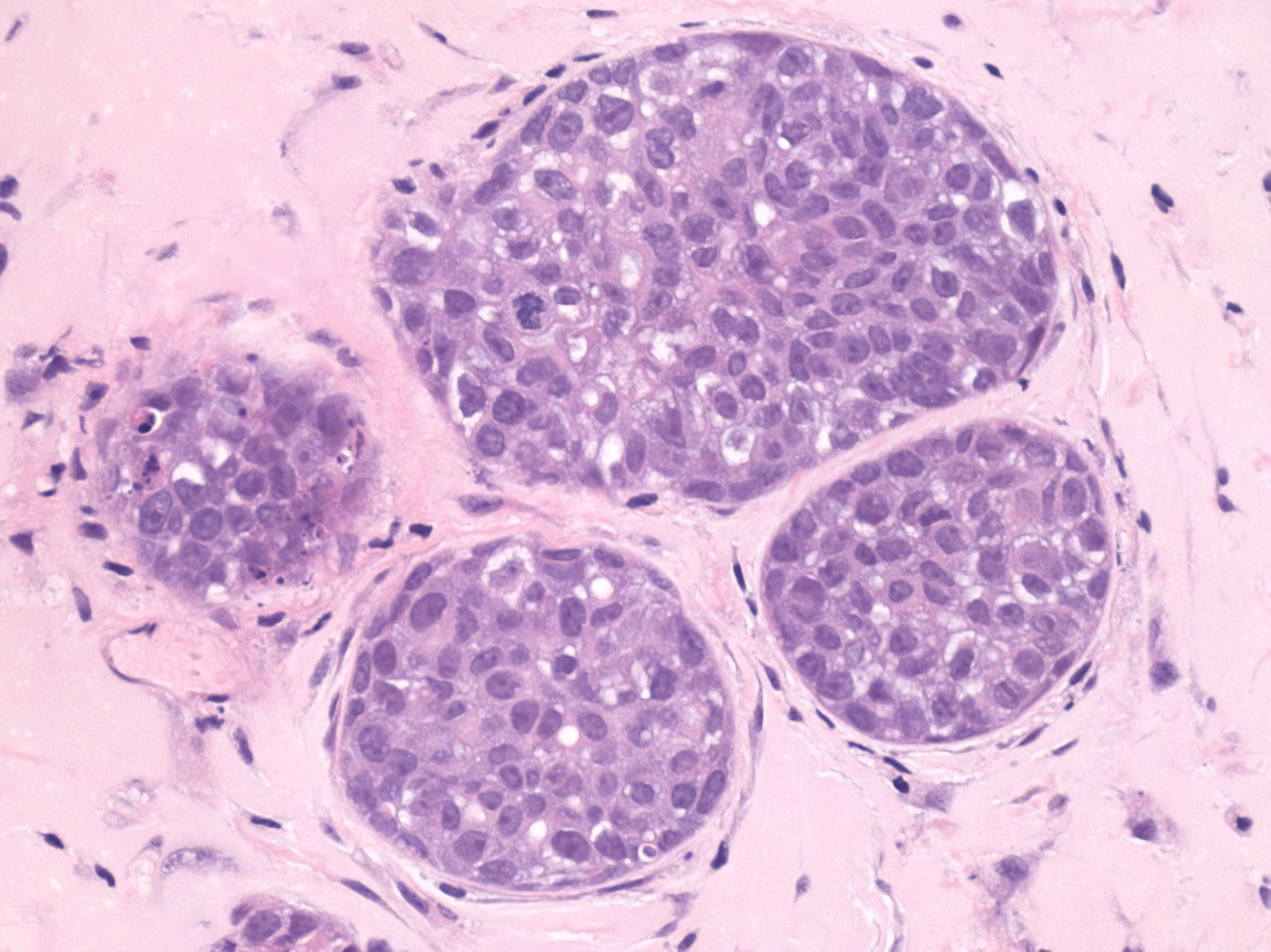

Supplement: Supplementary file 1 [file LSA-2019-00425_SdataF1A1.jpg]

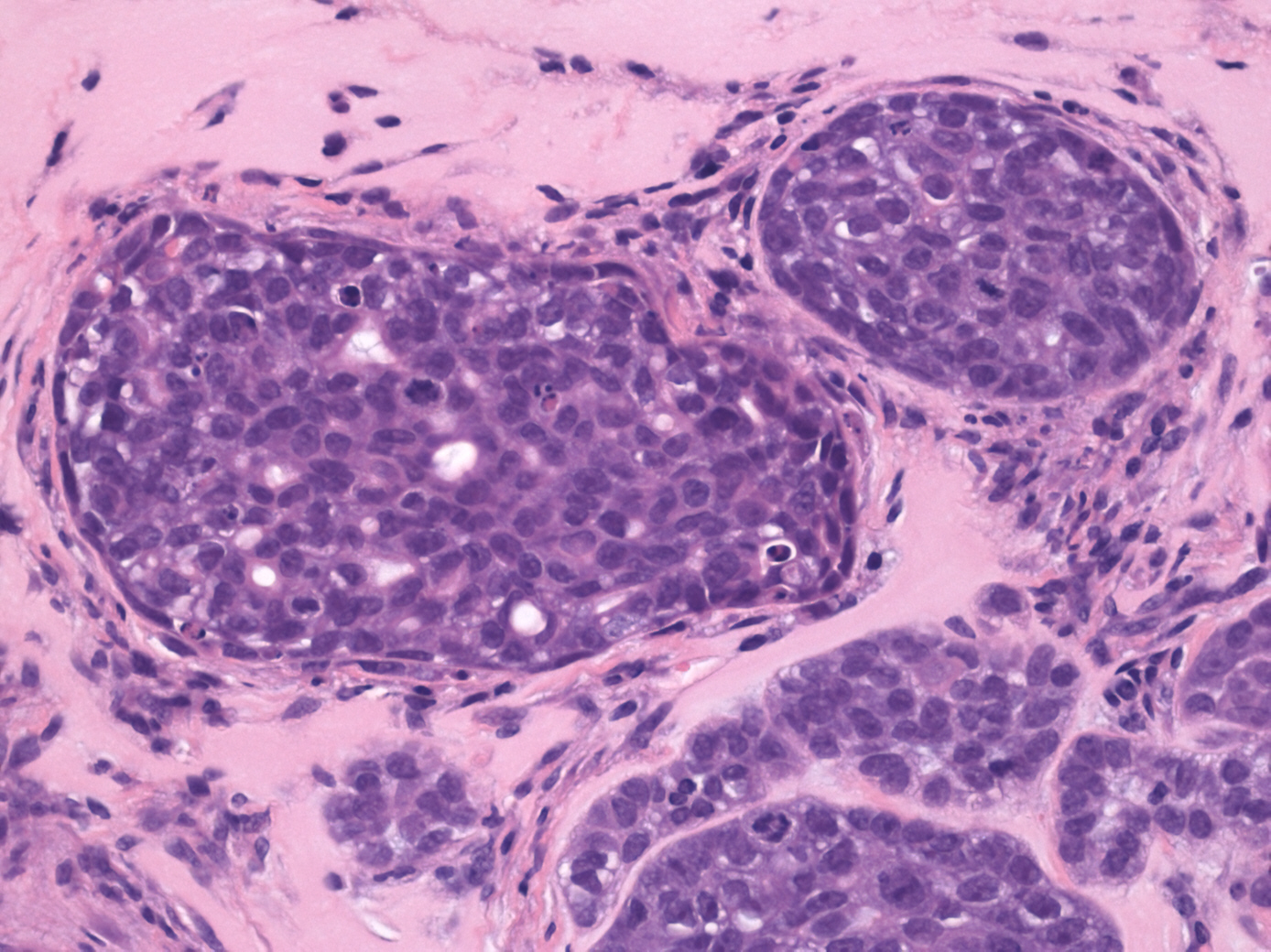

Supplement: Supplementary file 2 [file LSA-2019-00425_SdataF1A2.jpg]

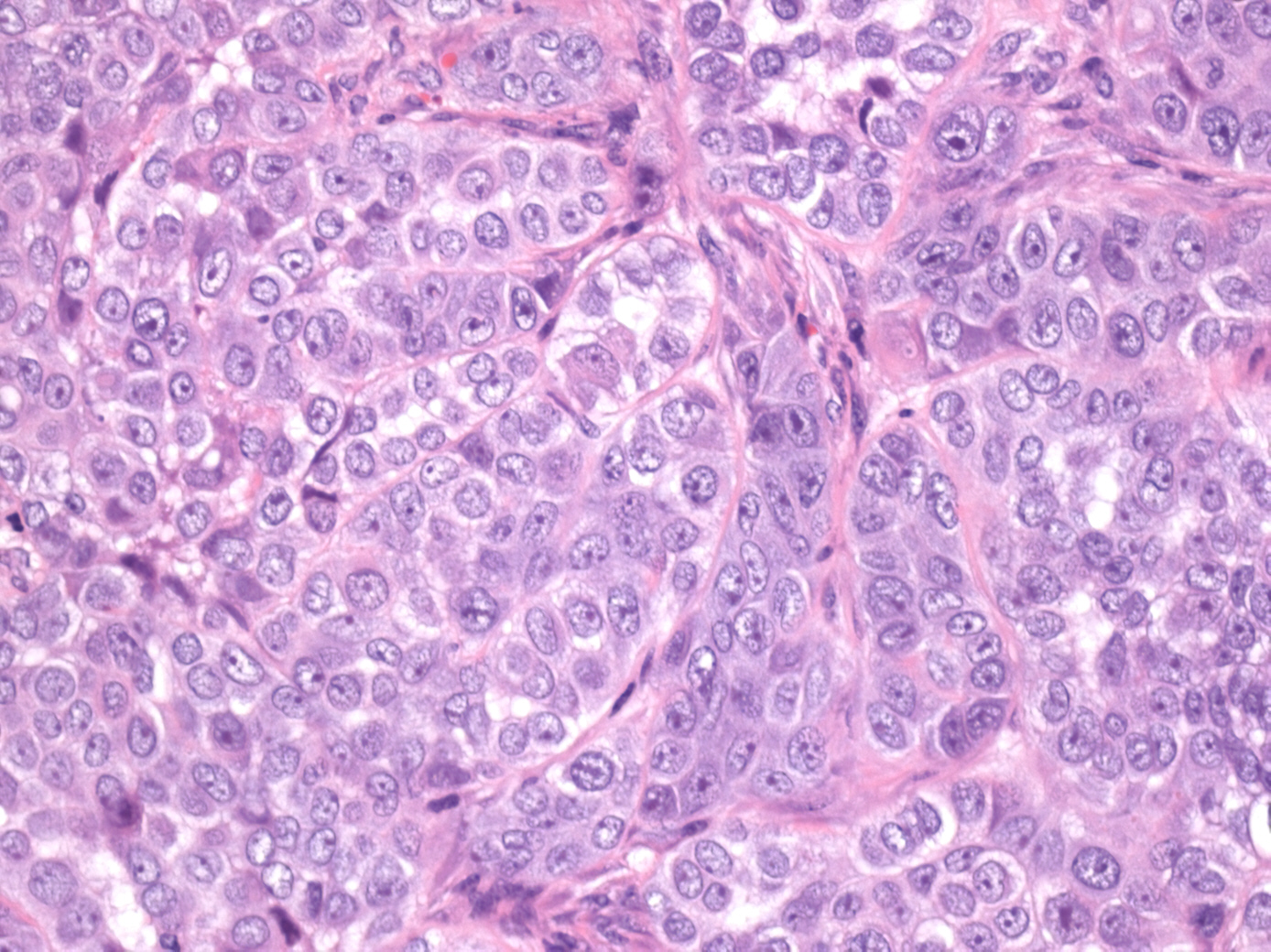

Supplement: Supplementary file 3 [file LSA-2019-00425_SdataF1A3.jpg]

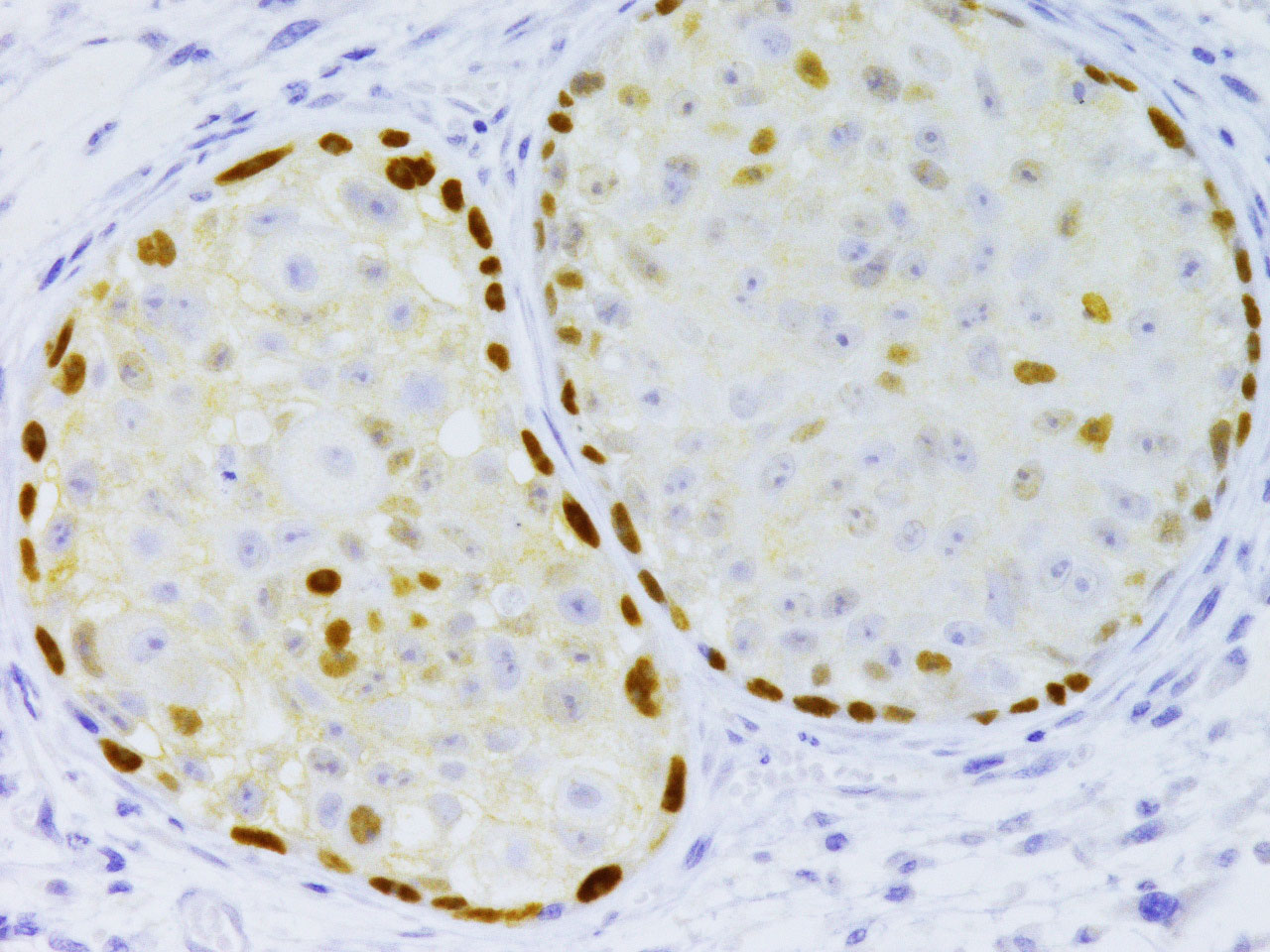

Supplement: Supplementary file 4 [file LSA-2019-00425_SdataF1A4.jpg]

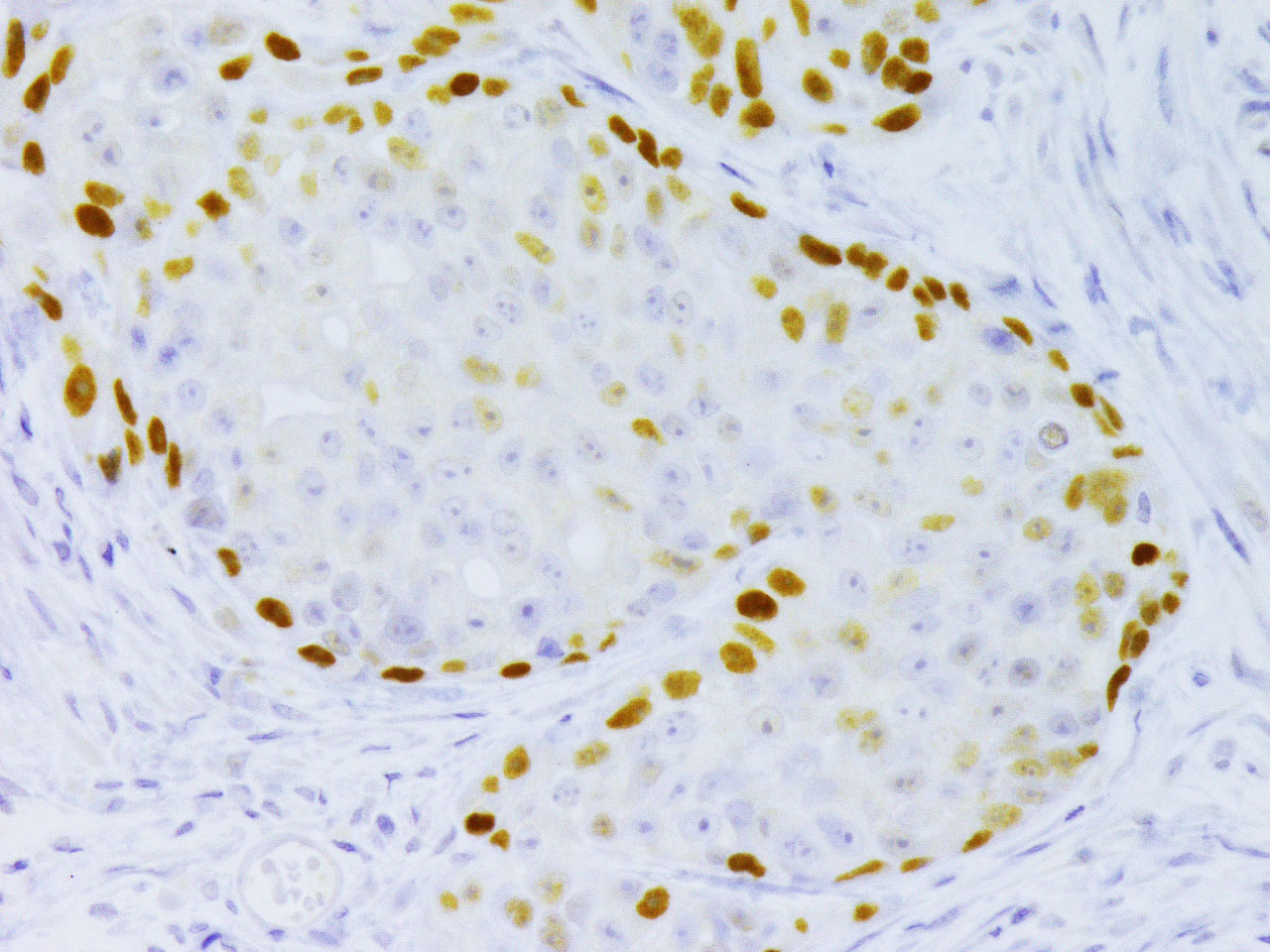

Supplement: Supplementary file 5 [file LSA-2019-00425_SdataF1A5.jpg]

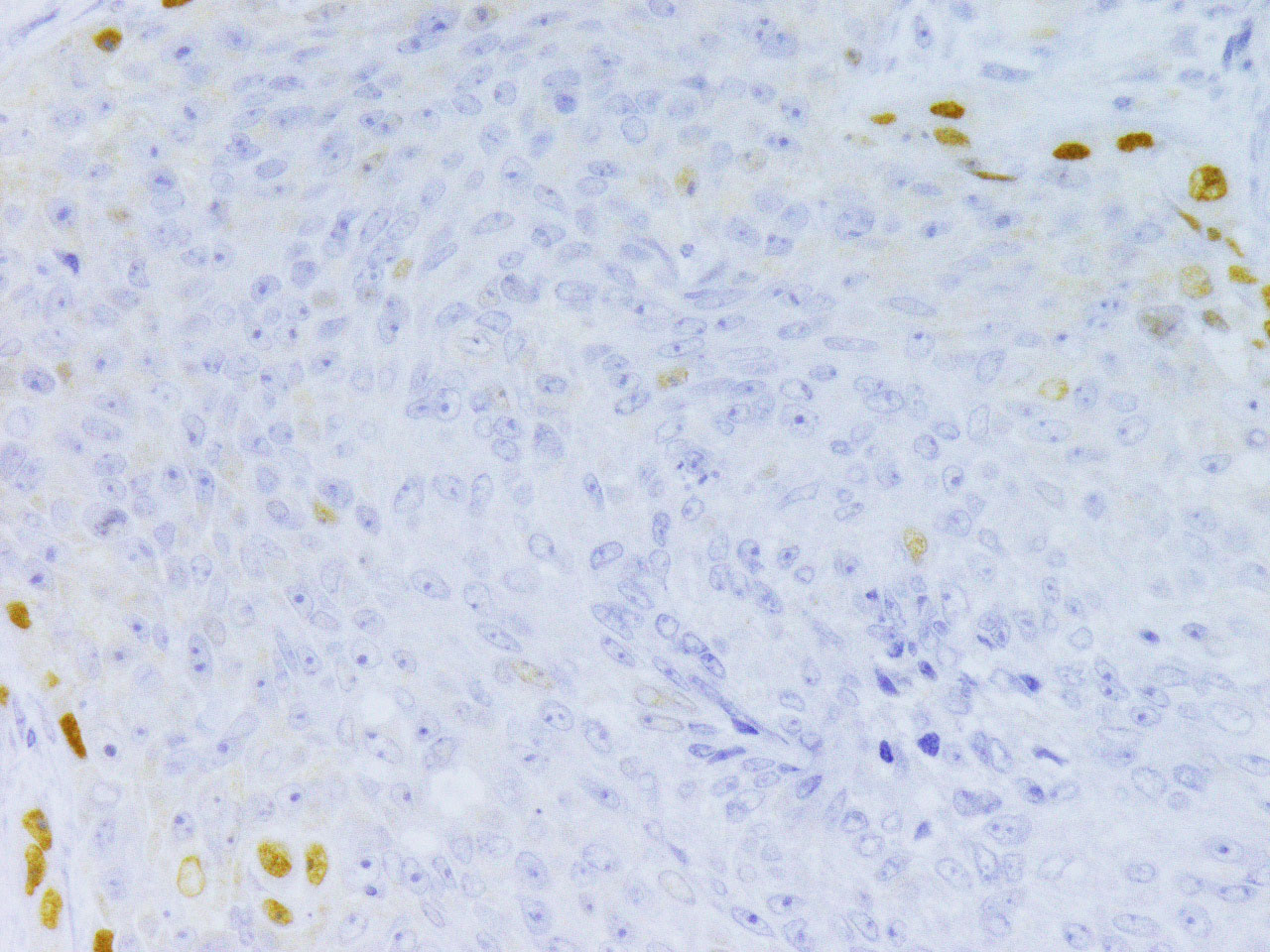

Supplement: Supplementary file 6 [file LSA-2019-00425_SdataF1A6.jpg]

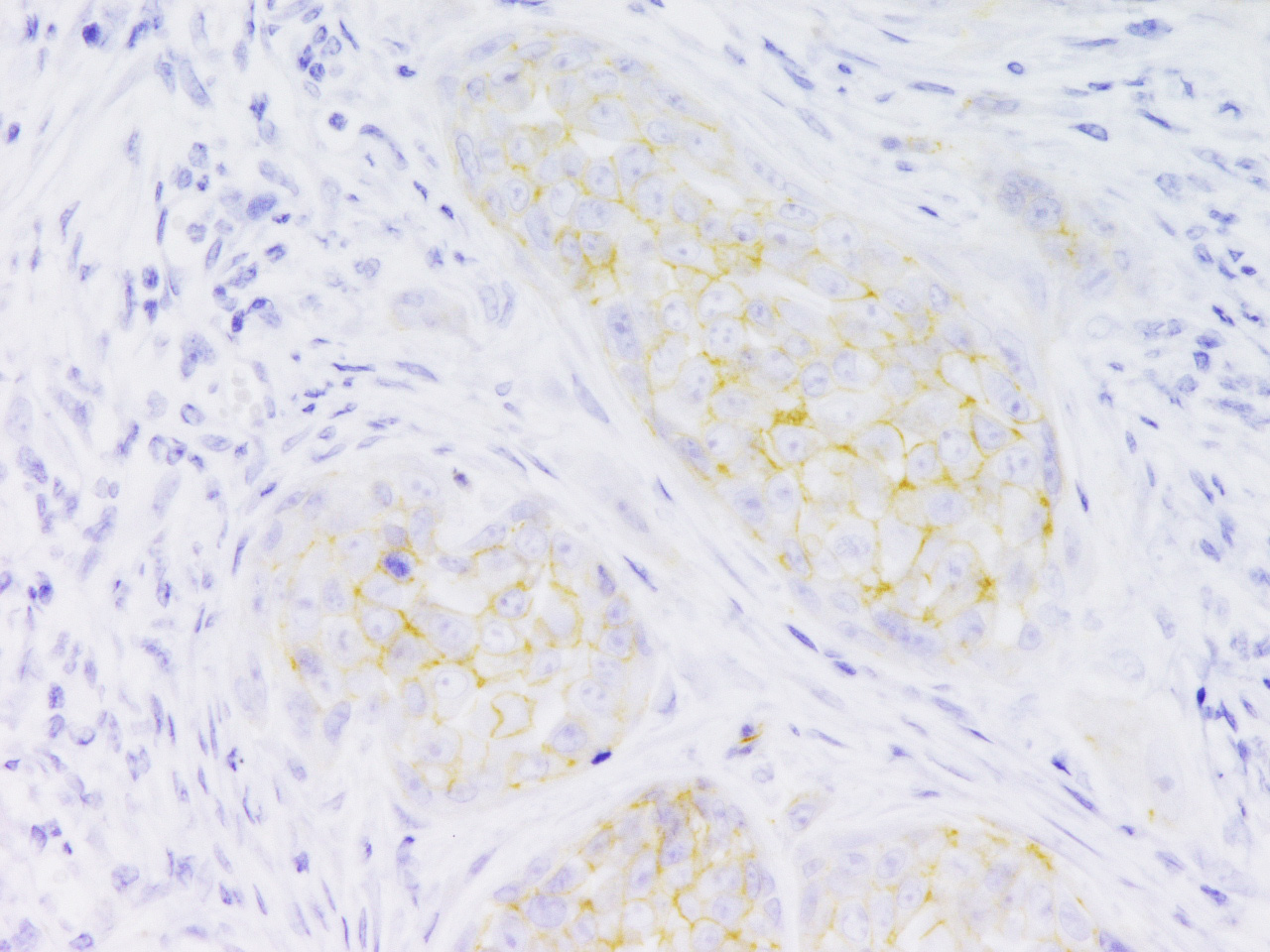

Supplement: Supplementary file 7 [file LSA-2019-00425_SdataF1A7.jpg]

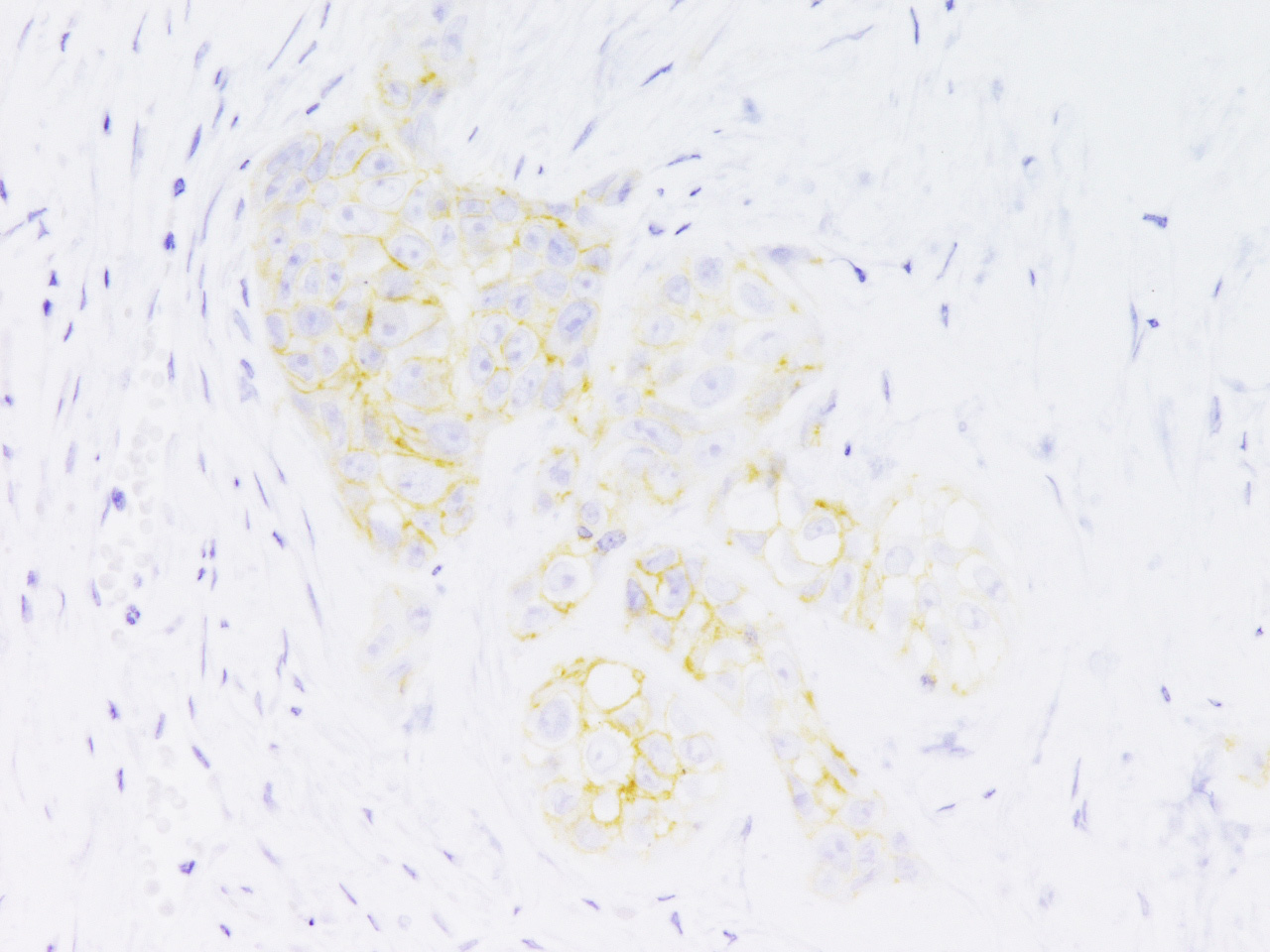

Supplement: Supplementary file 8 [file LSA-2019-00425_SdataF1A8.jpg]

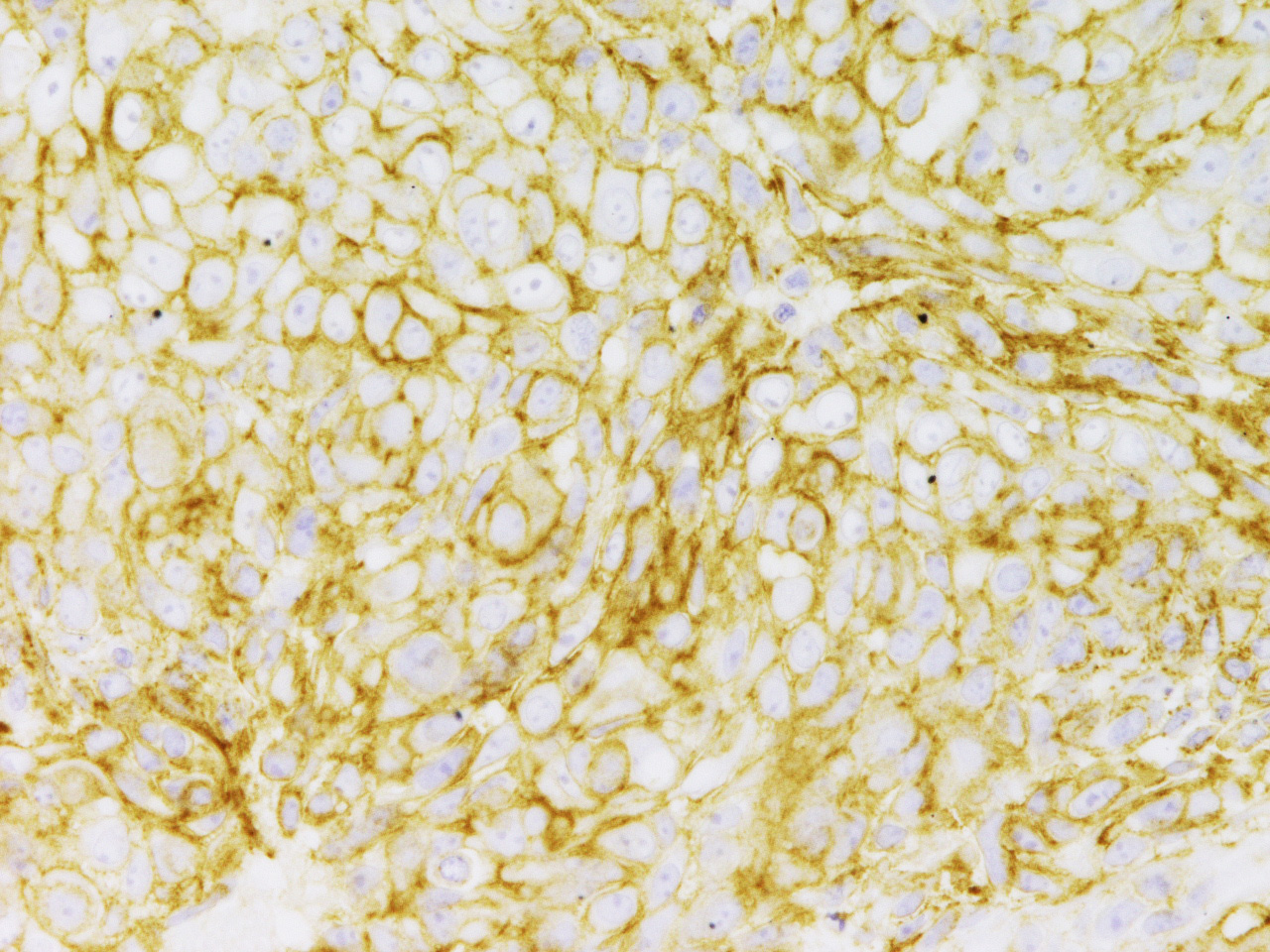

Supplement: Supplementary file 9 [file LSA-2019-00425_SdataF1A9.jpg]

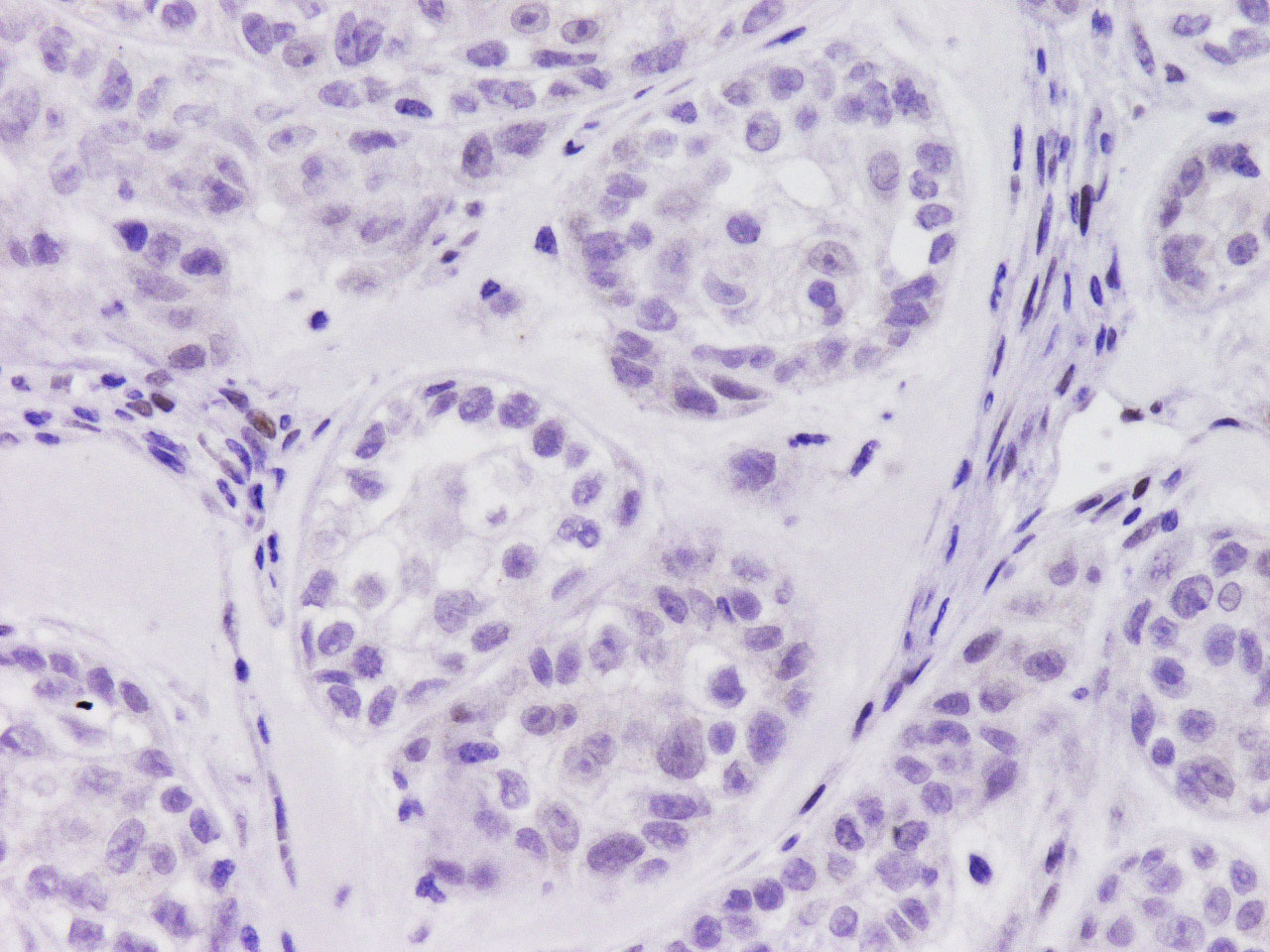

Supplement: Supplementary file 10 [file LSA-2019-00425_SdataF1A10.jpg]

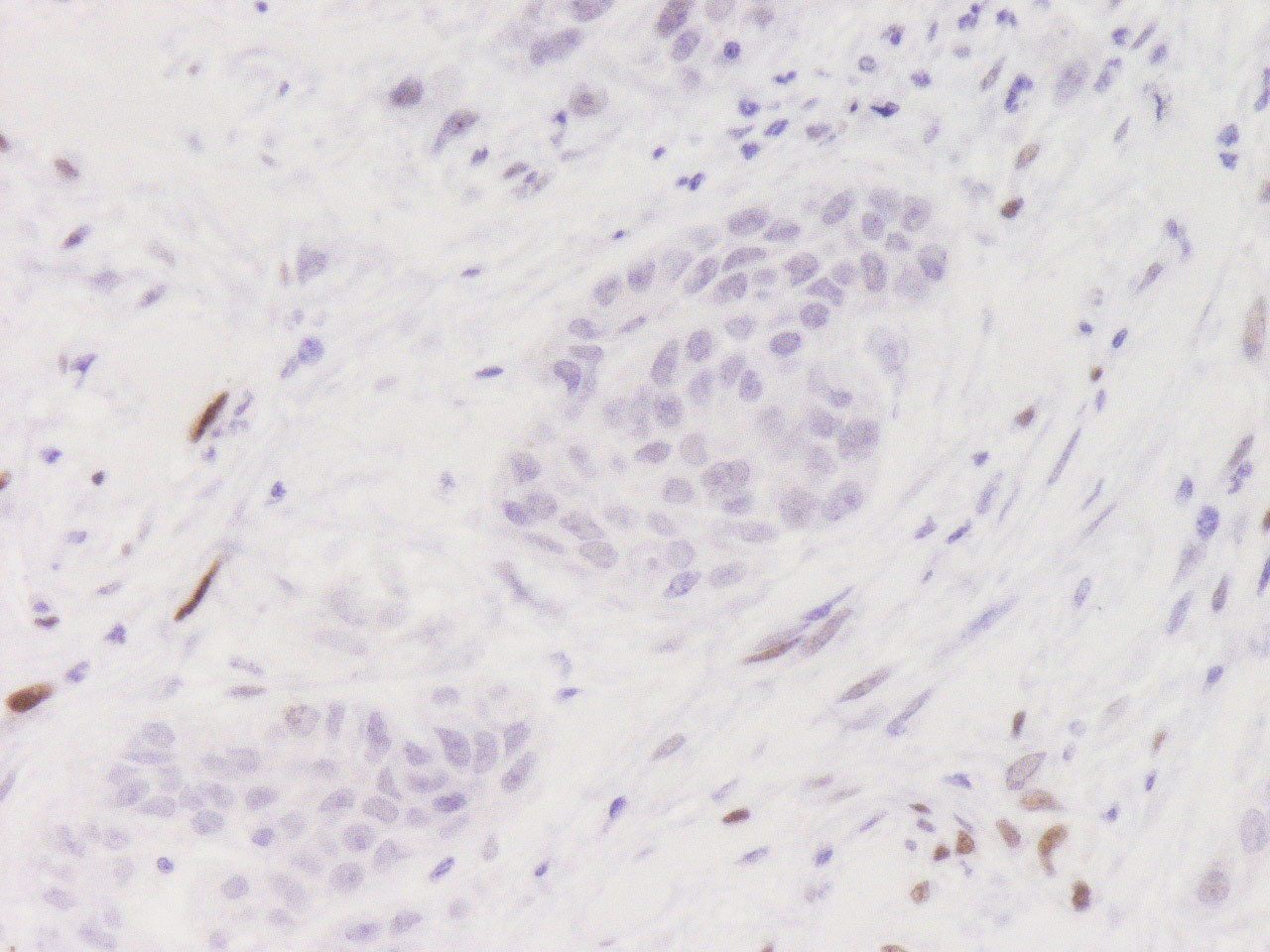

Supplement: Supplementary file 11 [file LSA-2019-00425_SdataF1A11.jpg]

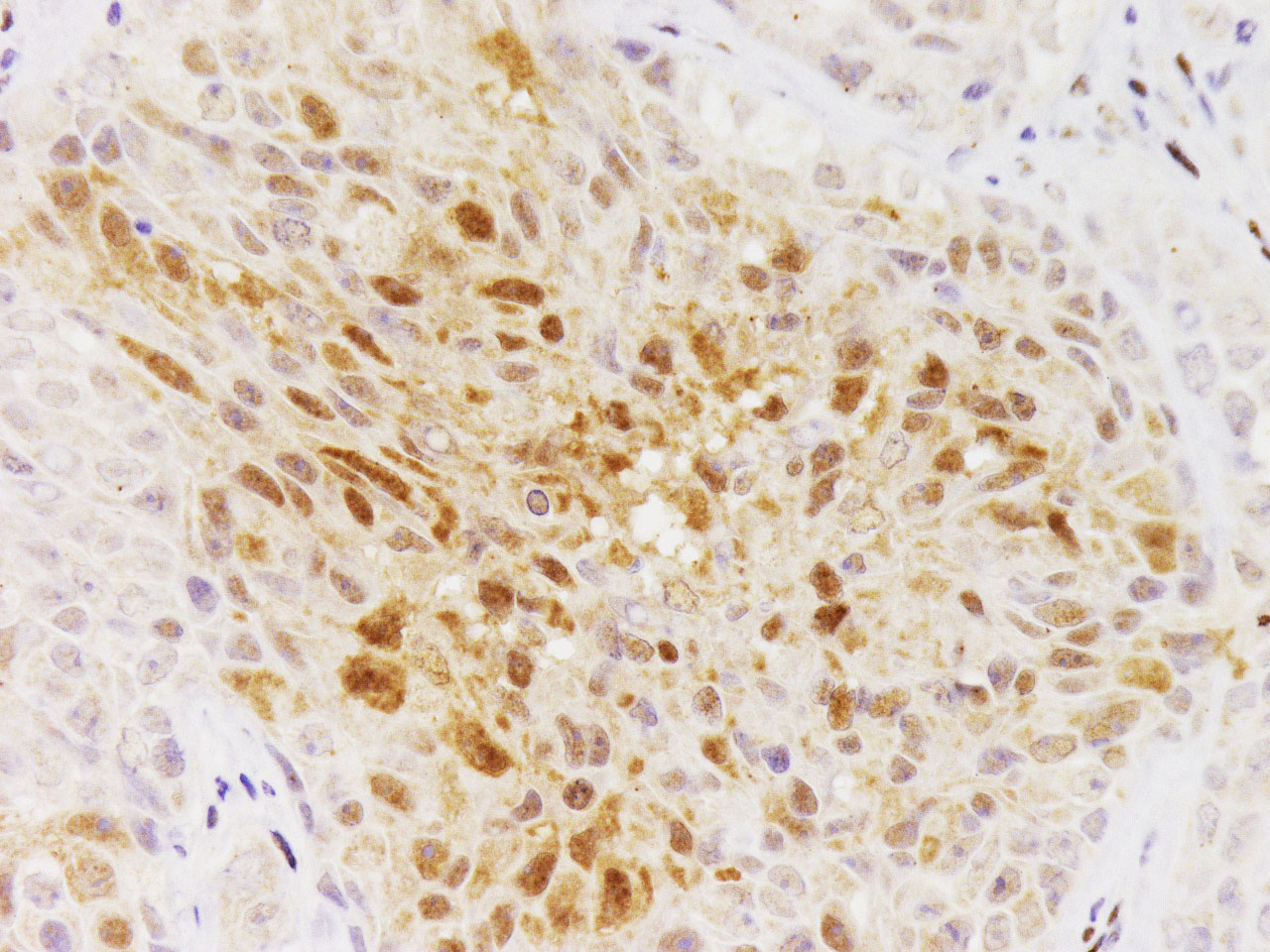

Supplement: Supplementary file 12 [file LSA-2019-00425_SdataF1A12.jpg]

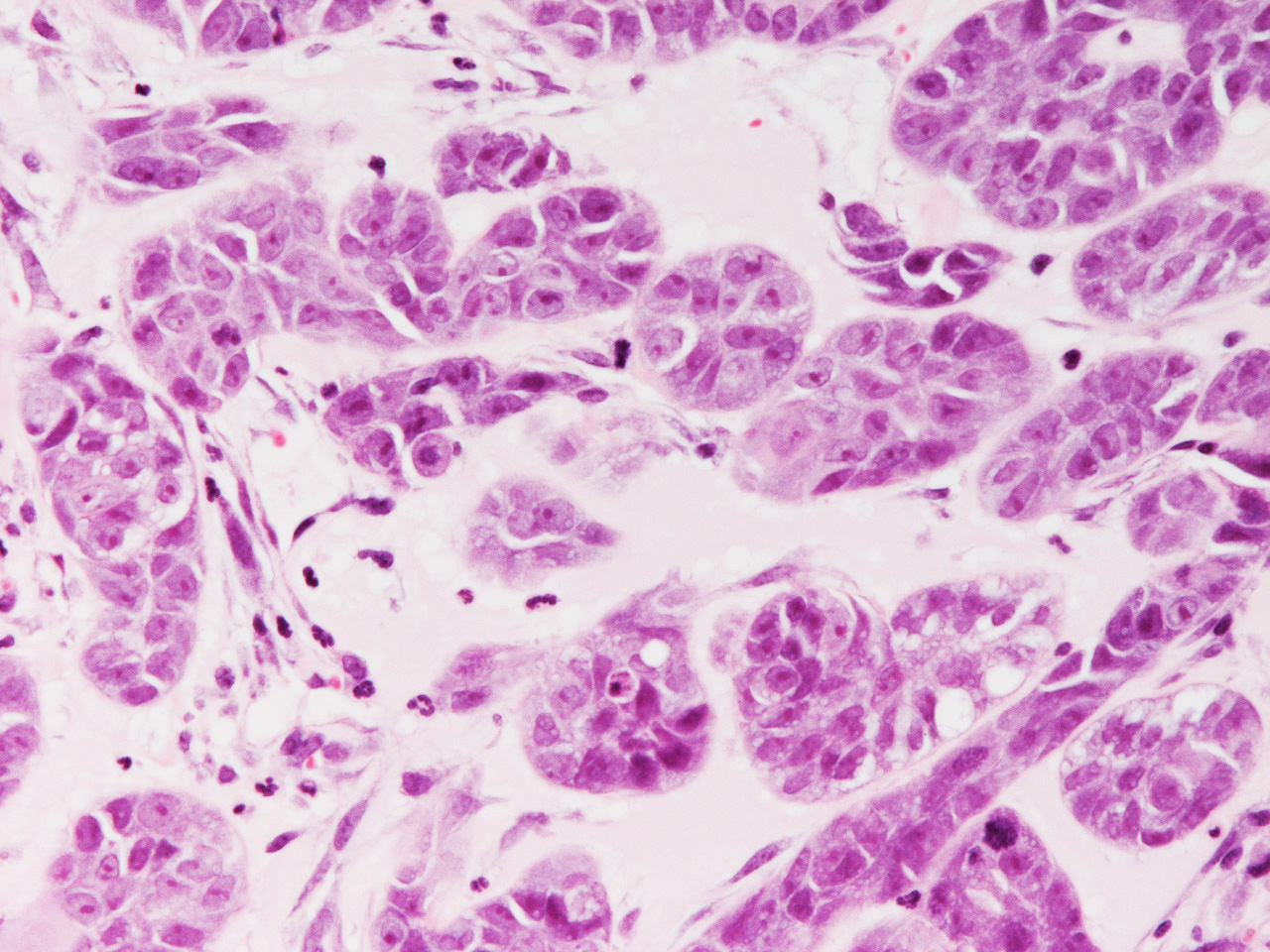

Supplement: Supplementary file 13 [file LSA-2019-00425_SdataF2A1.jpg]

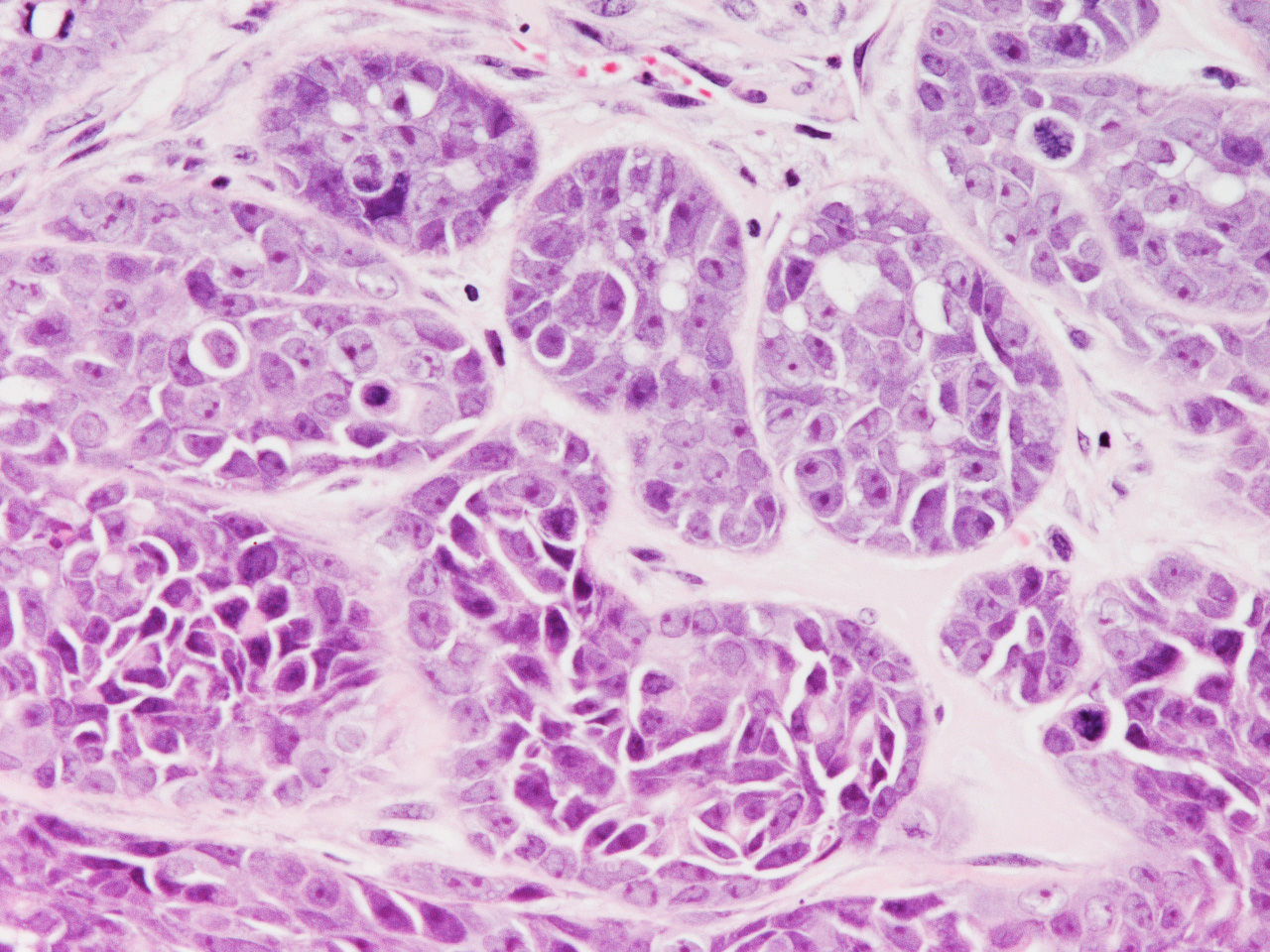

Supplement: Supplementary file 14 [file LSA-2019-00425_SdataF2A2.jpg]

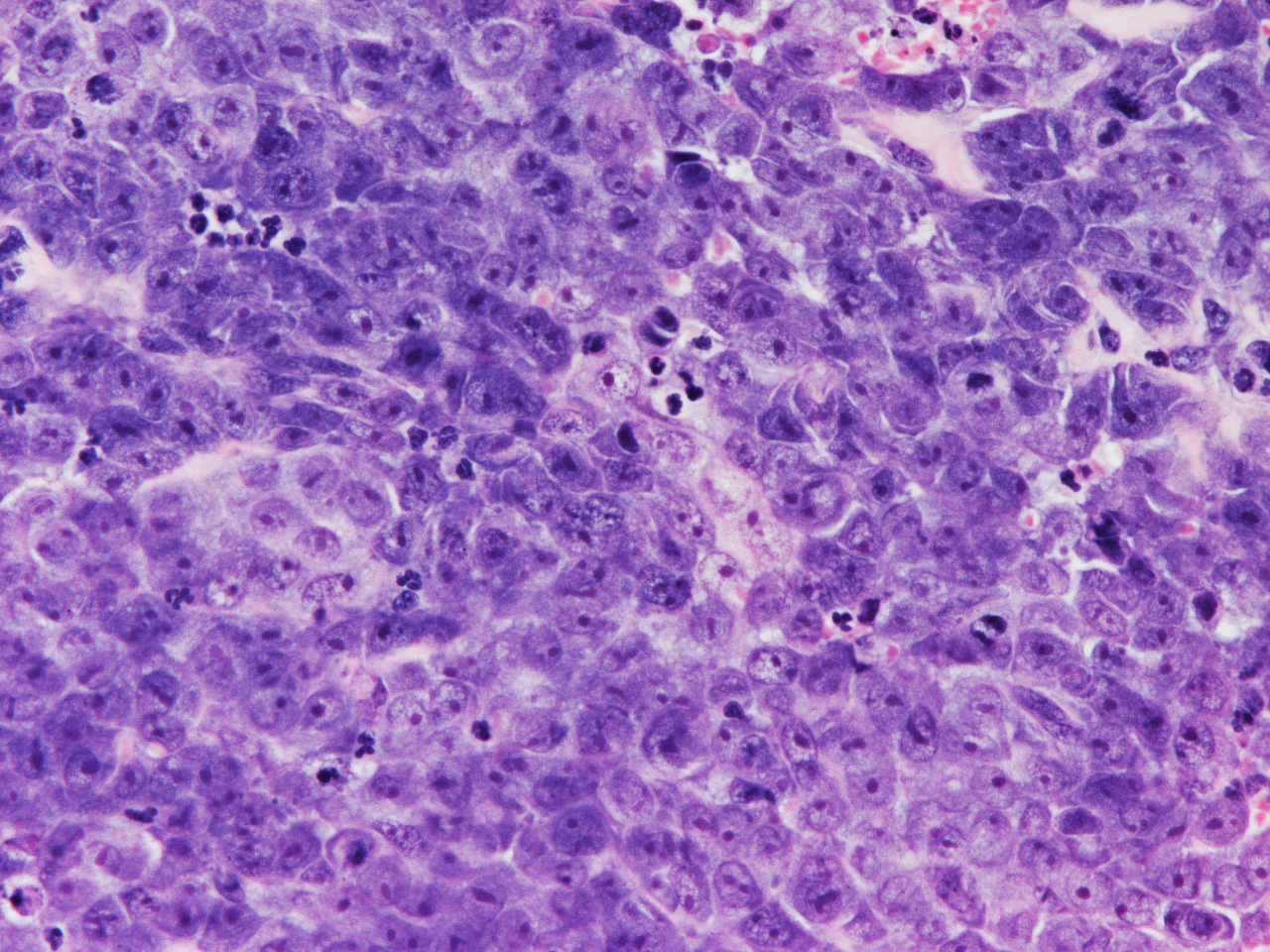

Supplement: Supplementary file 15 [file LSA-2019-00425_SdataF2A3.jpg]

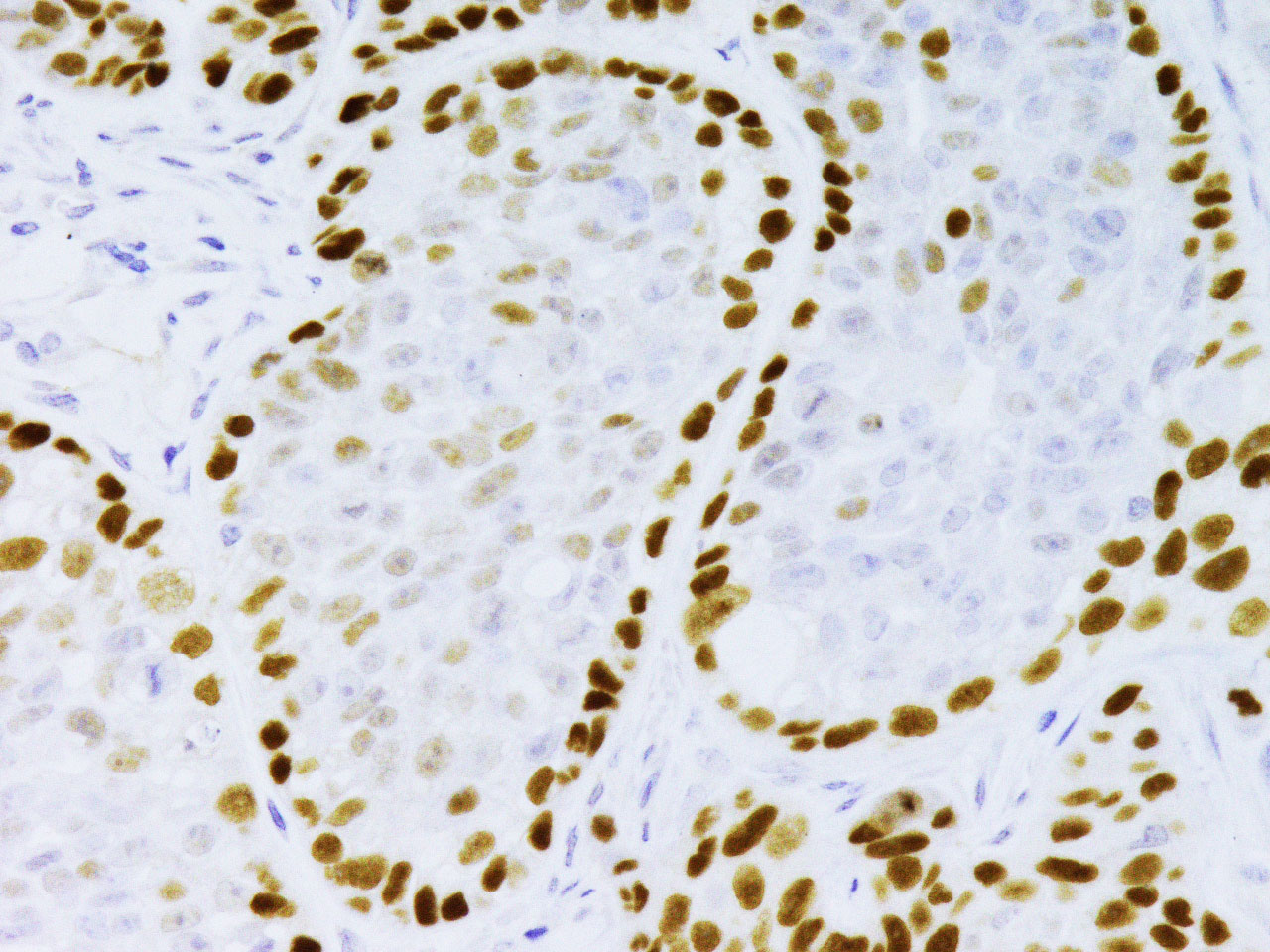

Supplement: Supplementary file 16 [file LSA-2019-00425_SdataF2A4.jpg]

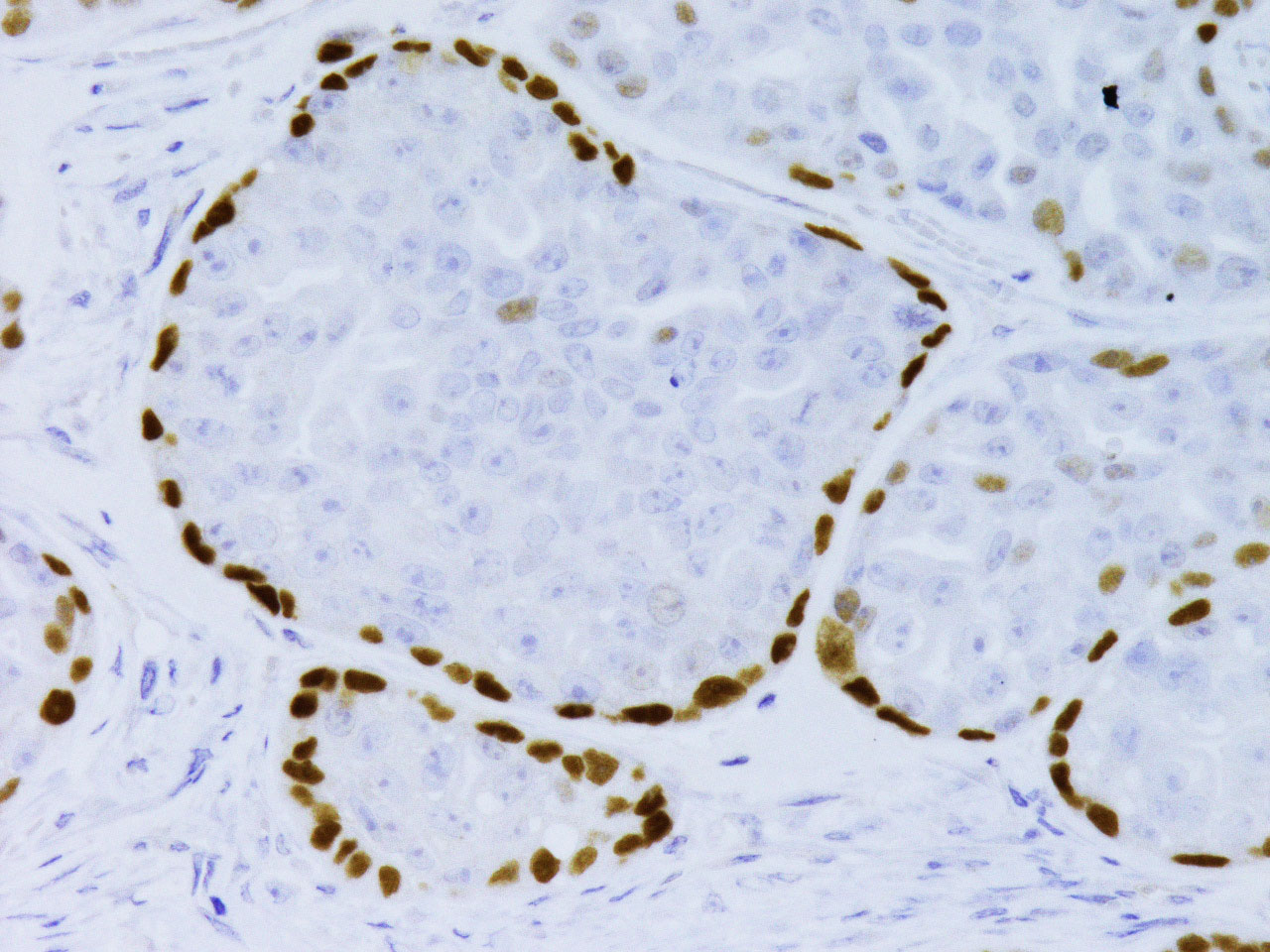

Supplement: Supplementary file 17 [file LSA-2019-00425_SdataF2A5.jpg]

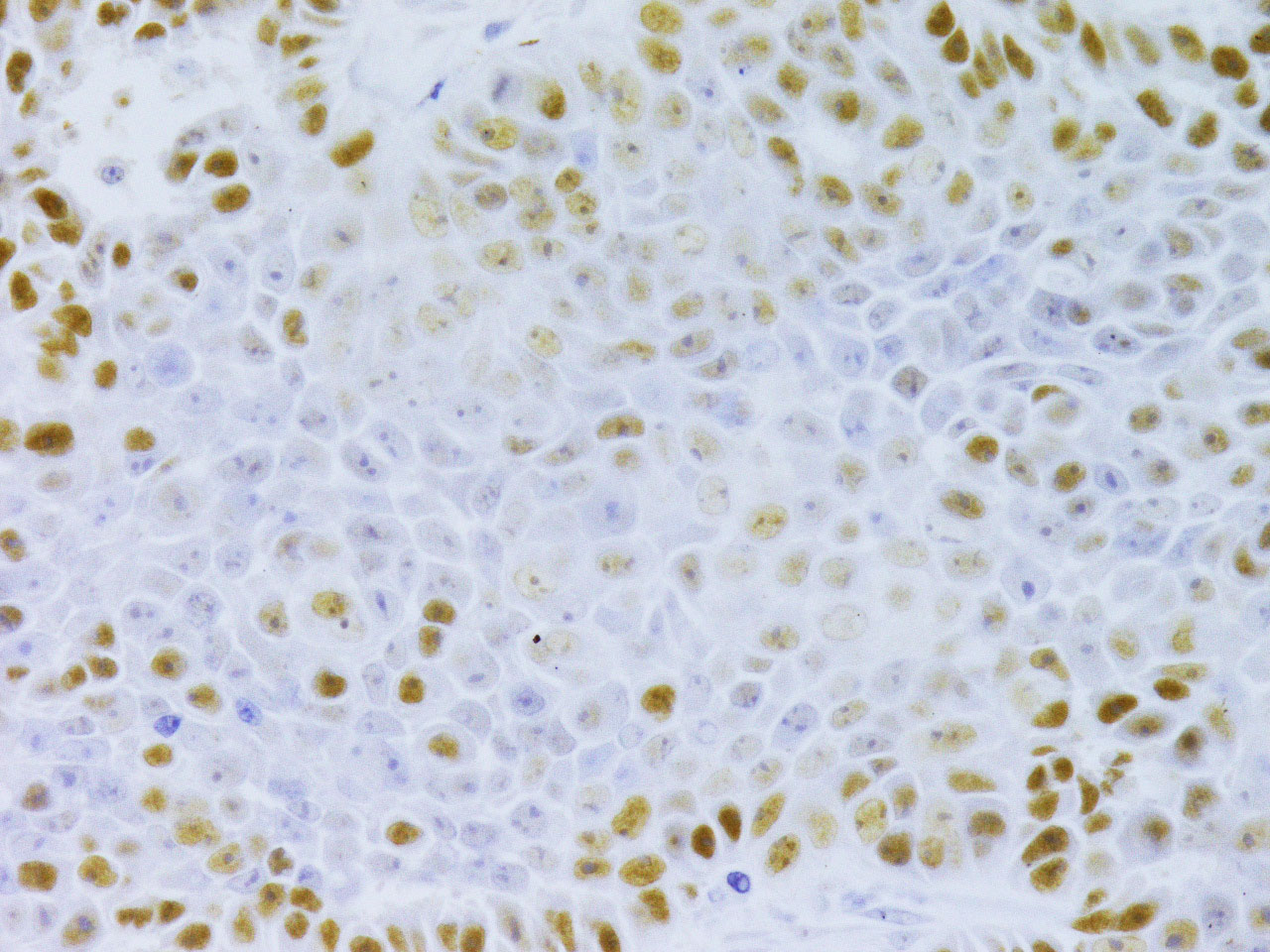

Supplement: Supplementary file 18 [file LSA-2019-00425_SdataF2A6.jpg]

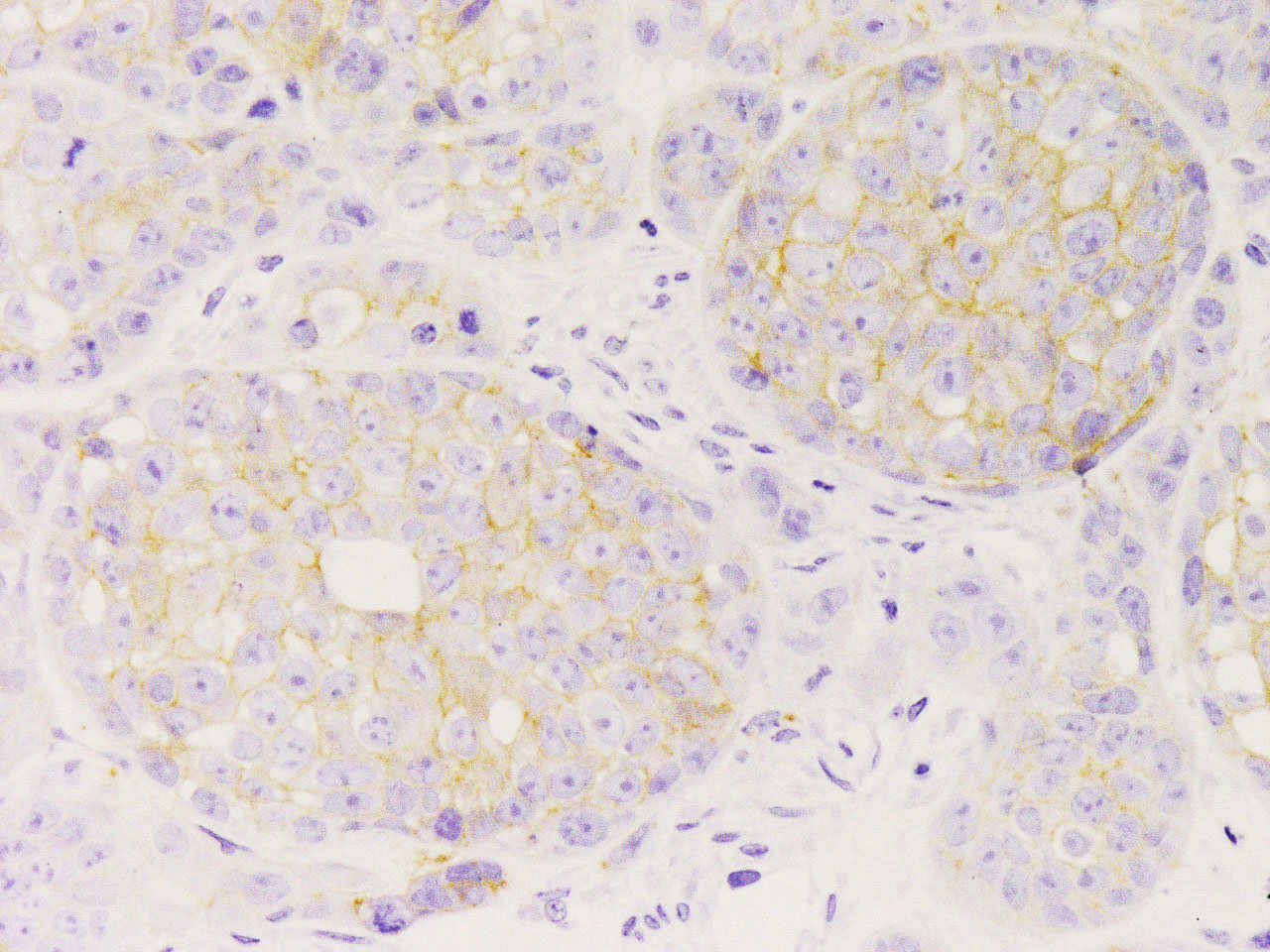

Supplement: Supplementary file 19 [file LSA-2019-00425_SdataF2A7.jpg]

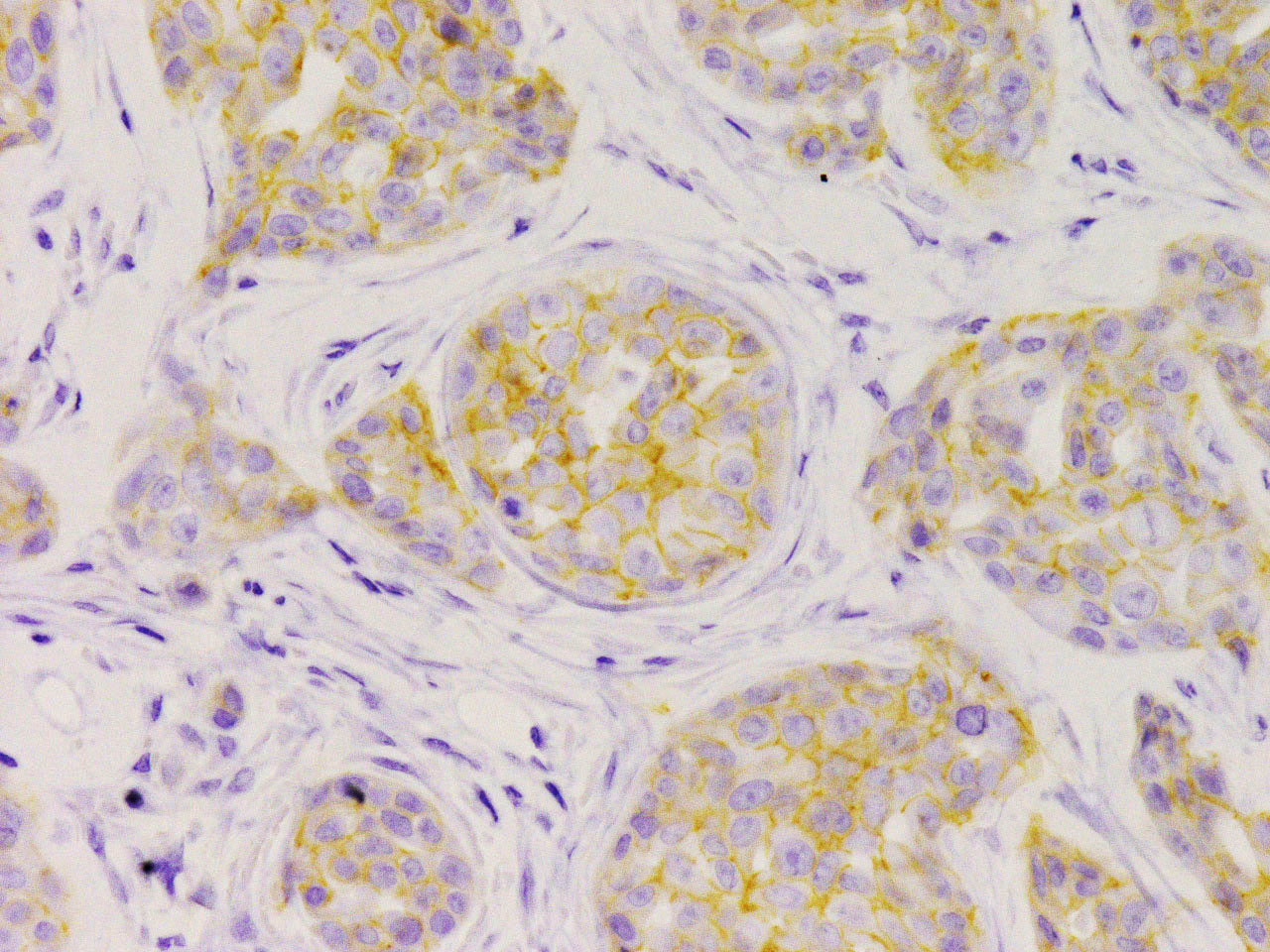

Supplement: Supplementary file 20 [file LSA-2019-00425_SdataF2A8.jpg]

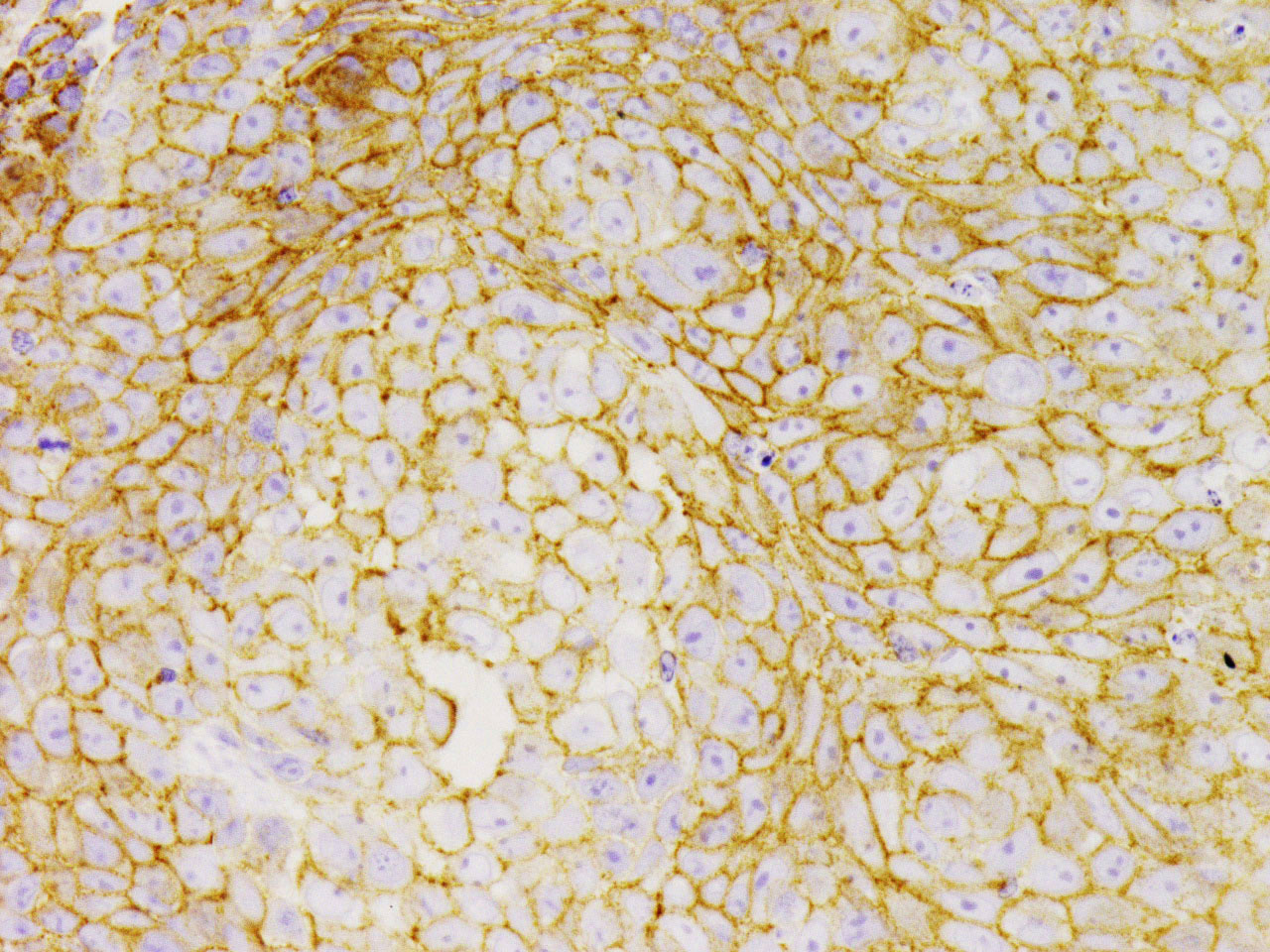

Supplement: Supplementary file 21 [file LSA-2019-00425_SdataF2A9.jpg]

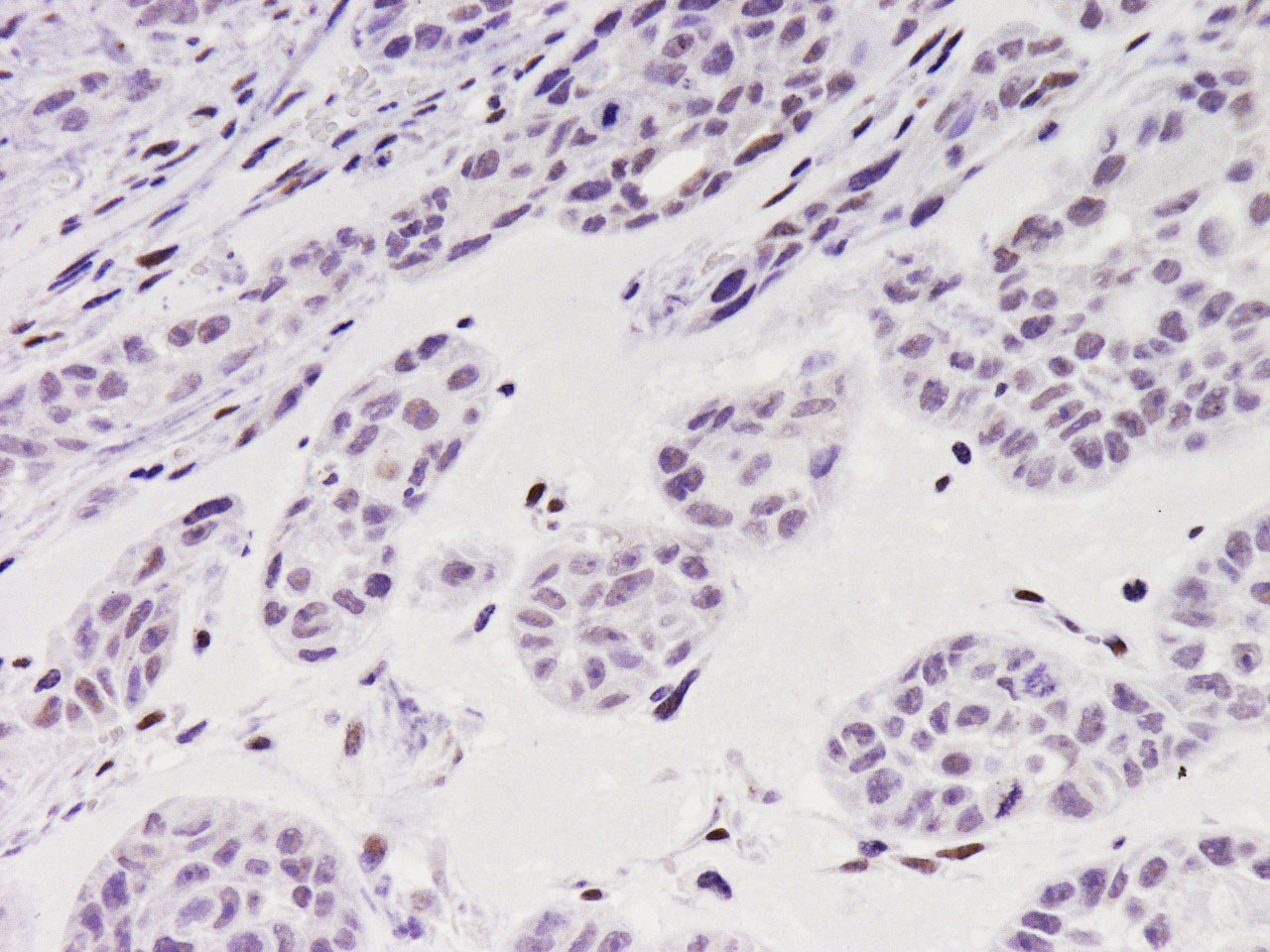

Supplement: Supplementary file 22 [file LSA-2019-00425_SdataF2A10.jpg]

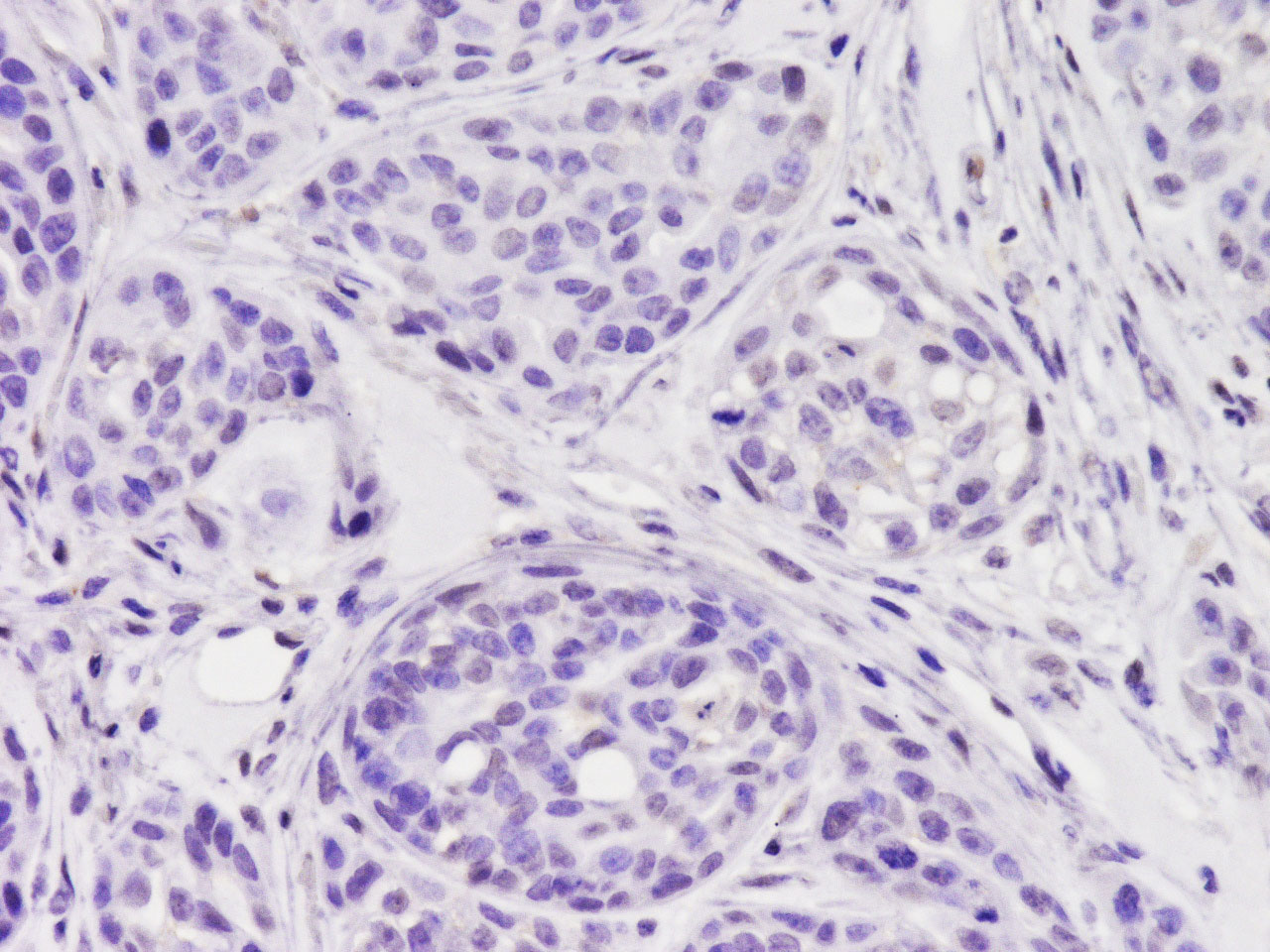

Supplement: Supplementary file 23 [file LSA-2019-00425_SdataF2A11.jpg]

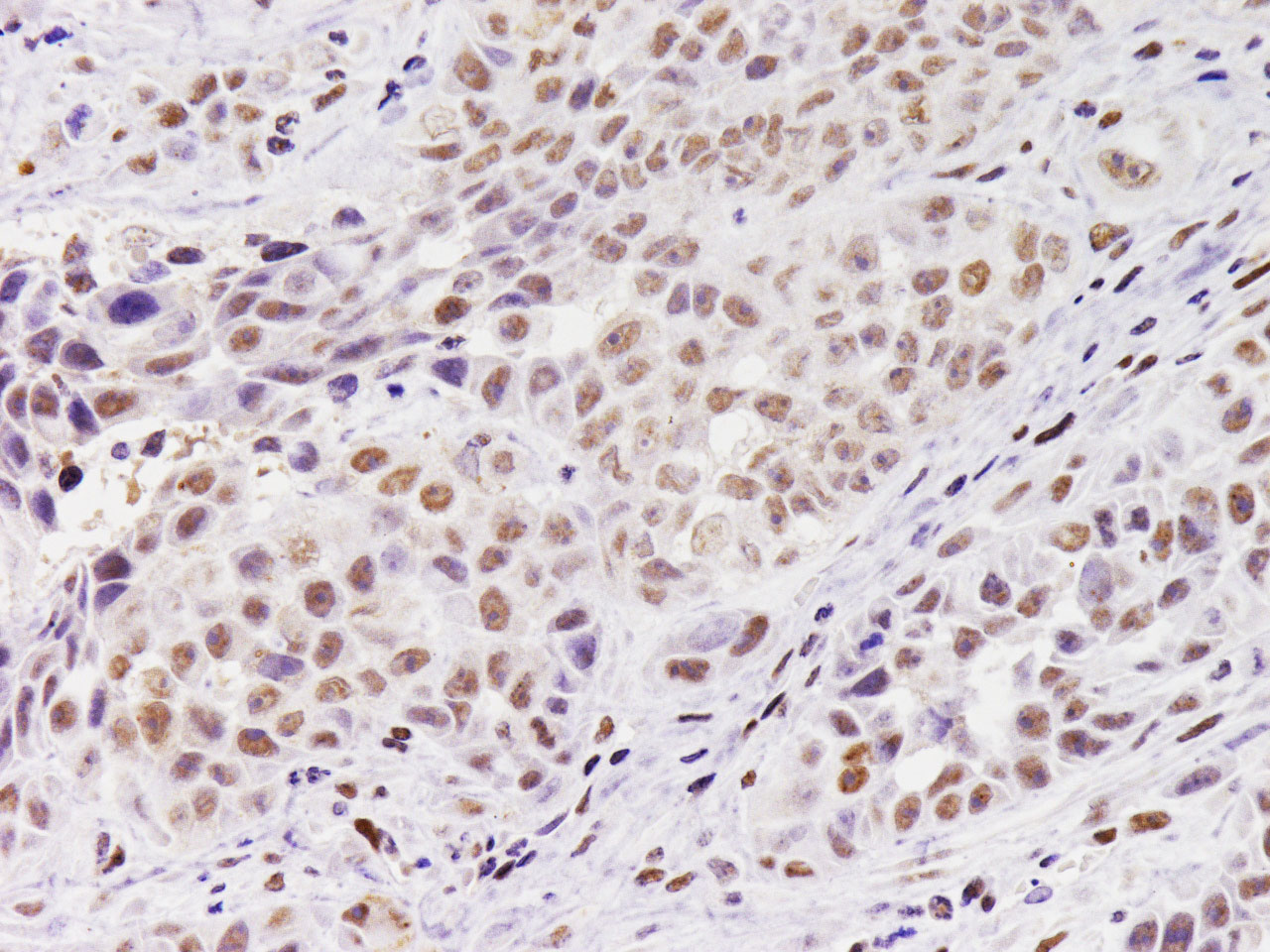

Supplement: Supplementary file 24 [file LSA-2019-00425_SdataF2A12.jpg]
