# Supplementary material for: Stromal fibroblasts induce metastatic tumor cell clusters via epithelial–mesenchymal plasticity
Source: Life Sci Alliance. 2019 Jul 22;2(4):e201900425. doi: 10.26508/lsa.201900425 (PMC6653778; doi:10.26508/lsa.201900425)

Full unedited gel for Figure 3C

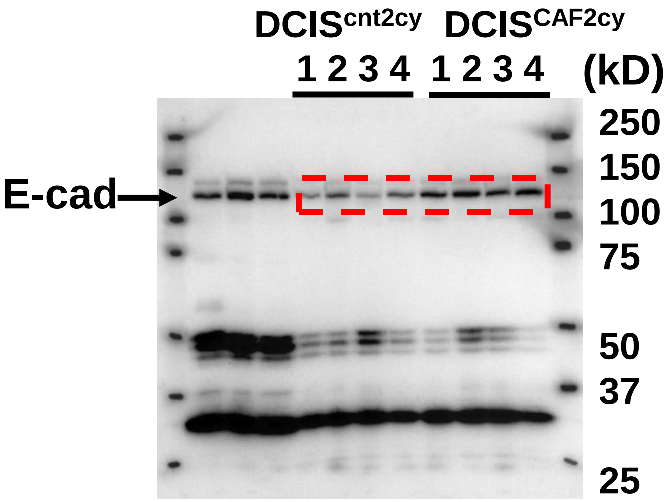

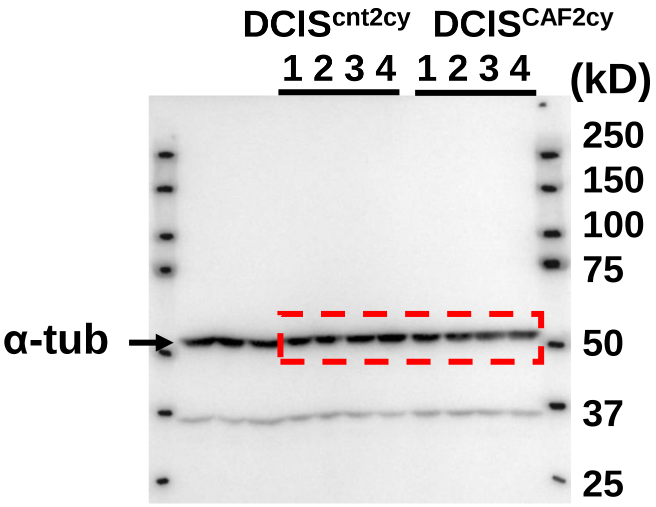

Full unedited gel for Figure 3D

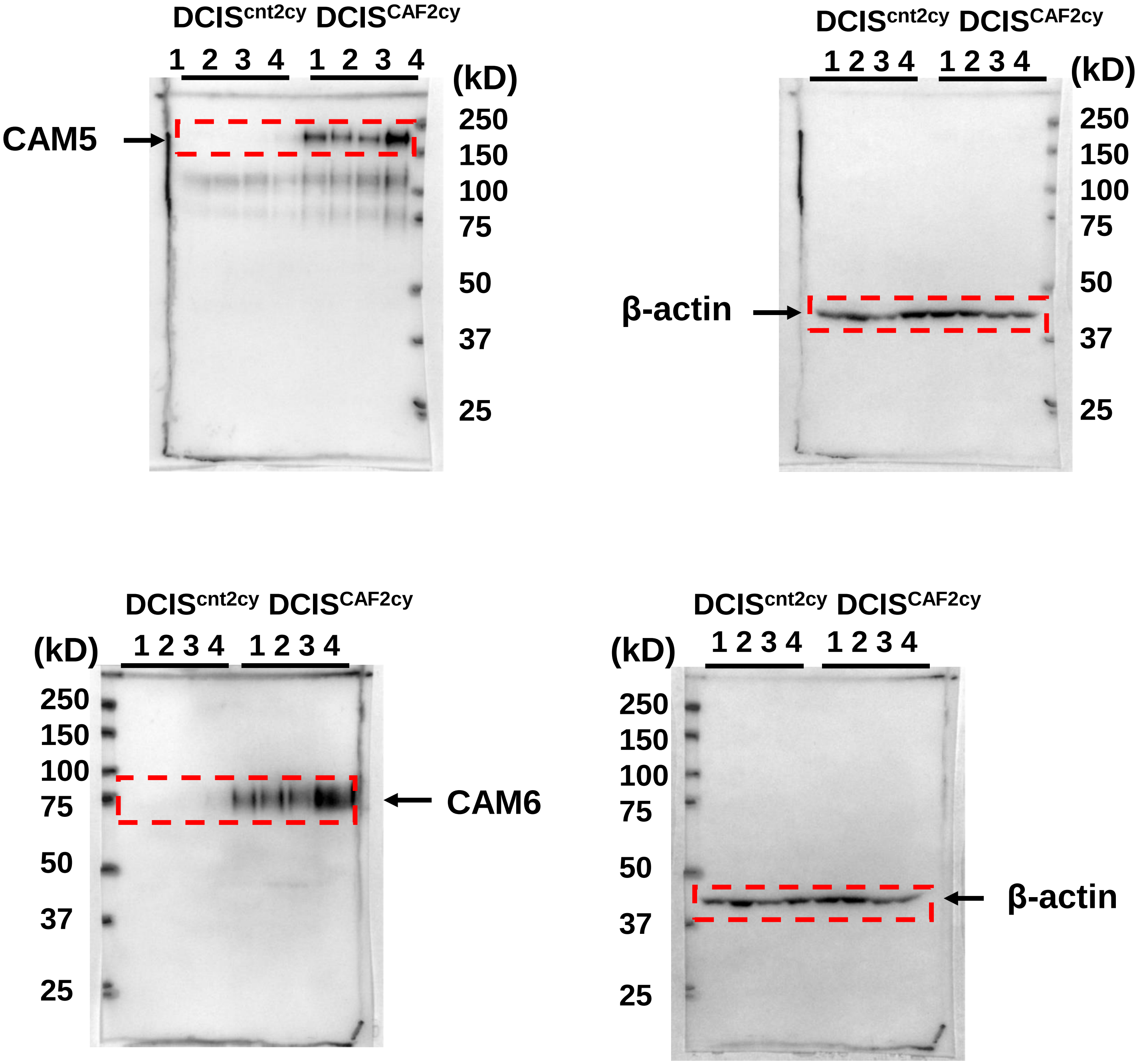

Full unedited gel for Figure 3E

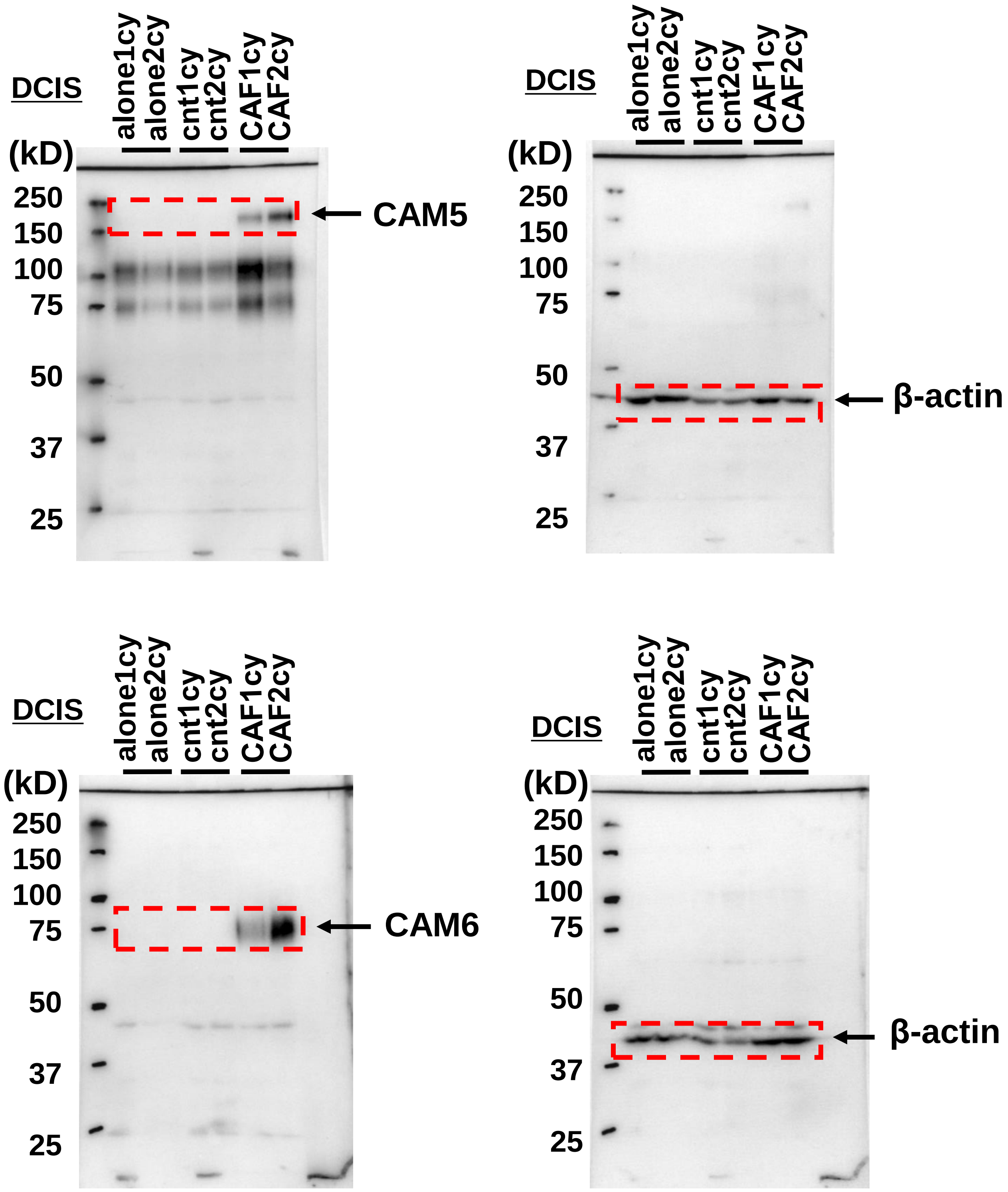

Full unedited gel for Figure 3H (top)

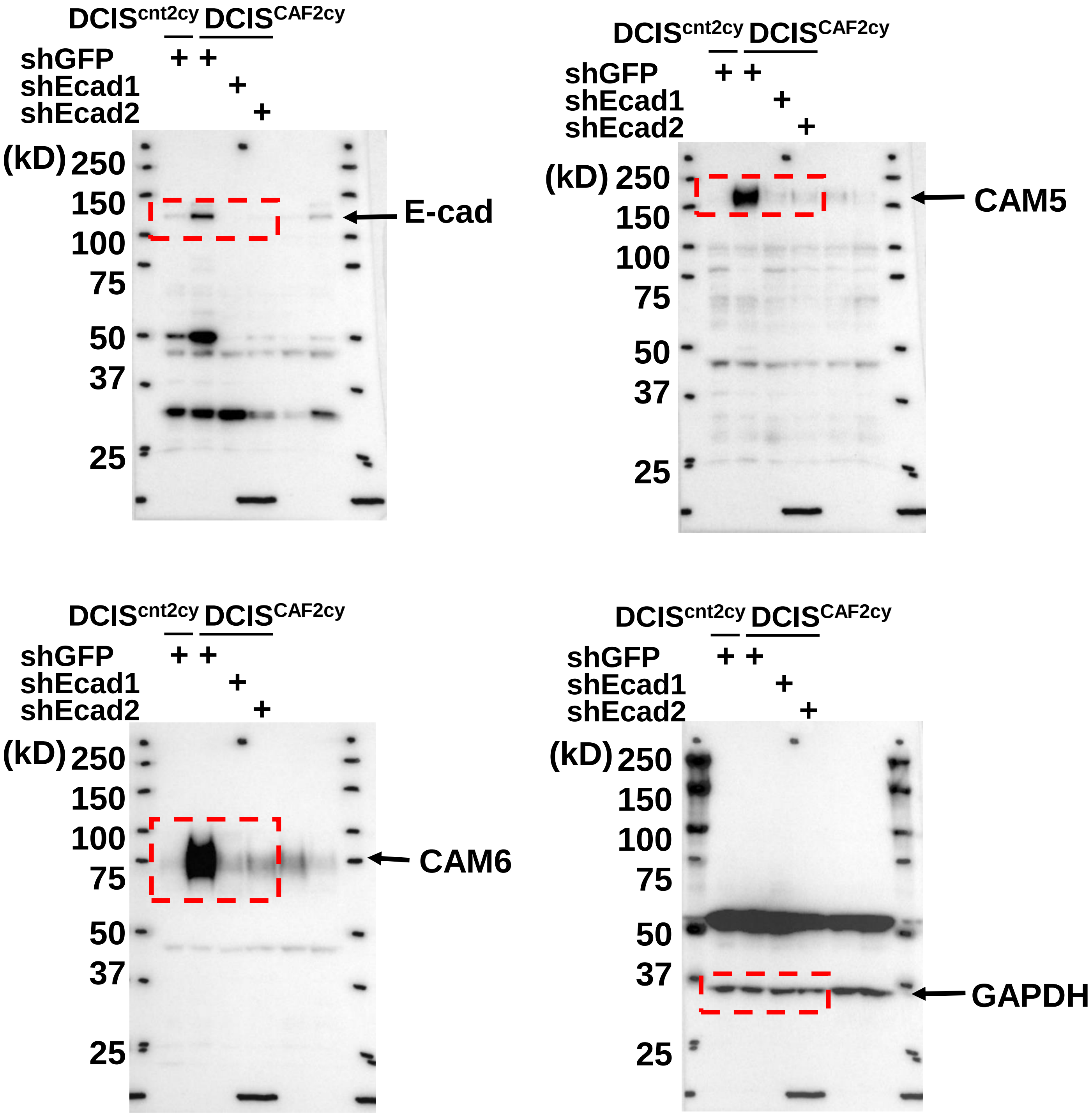

Full unedited gel for Figure 3H (down)

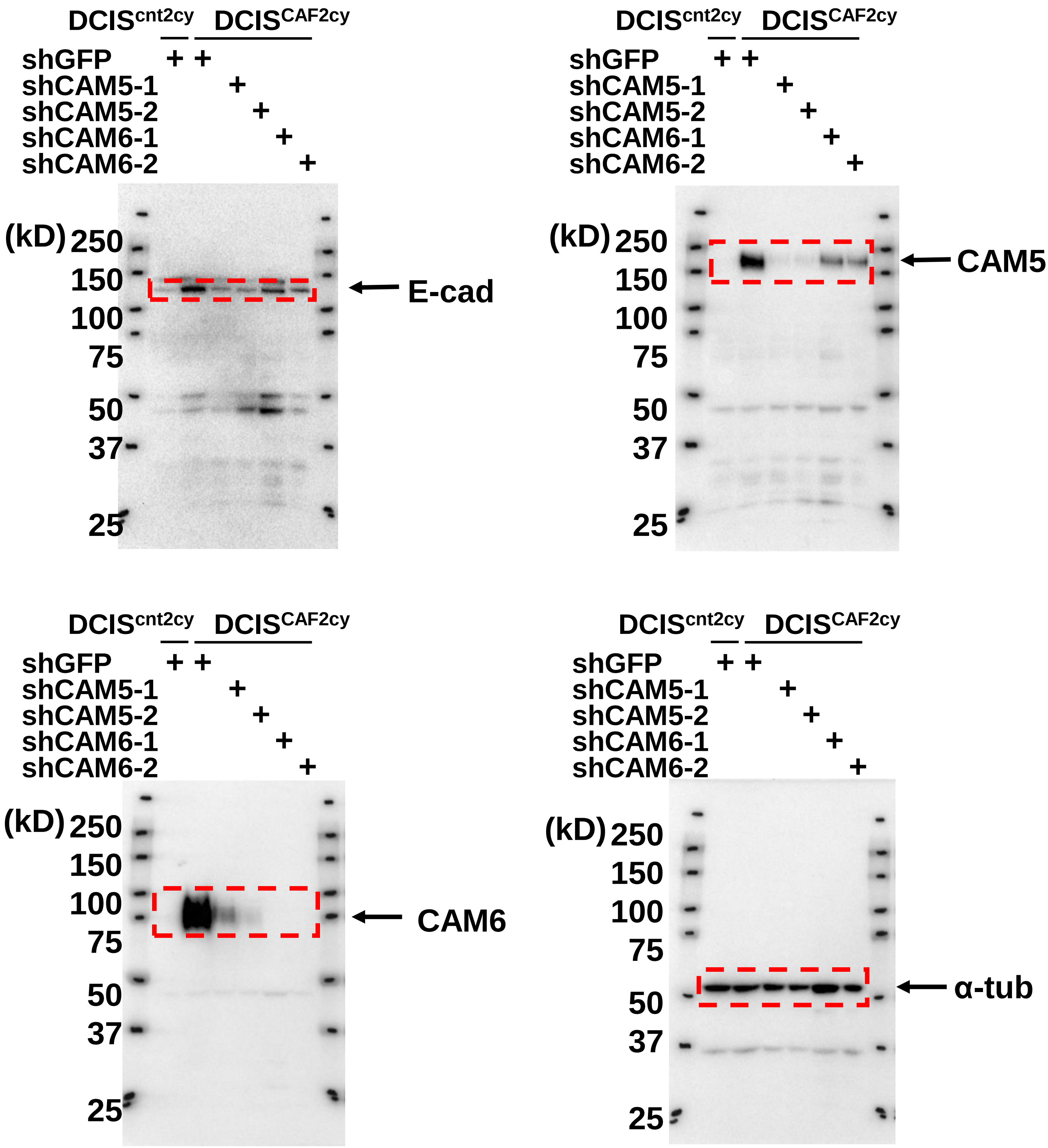

Full unedited gel for Figure 5A

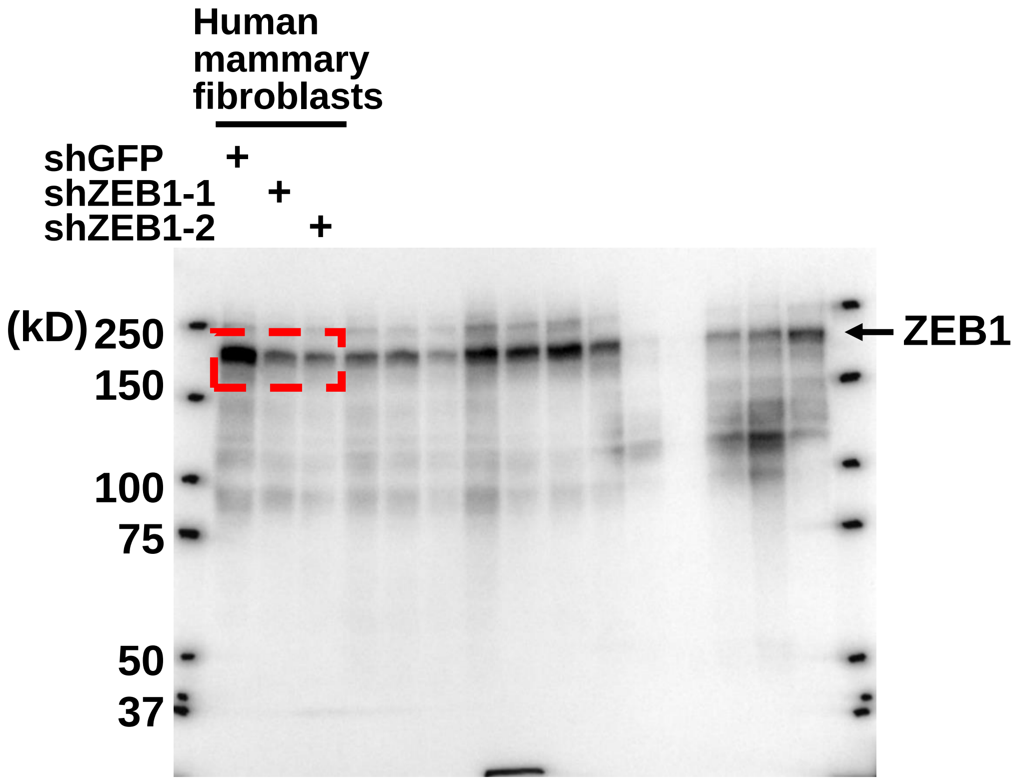

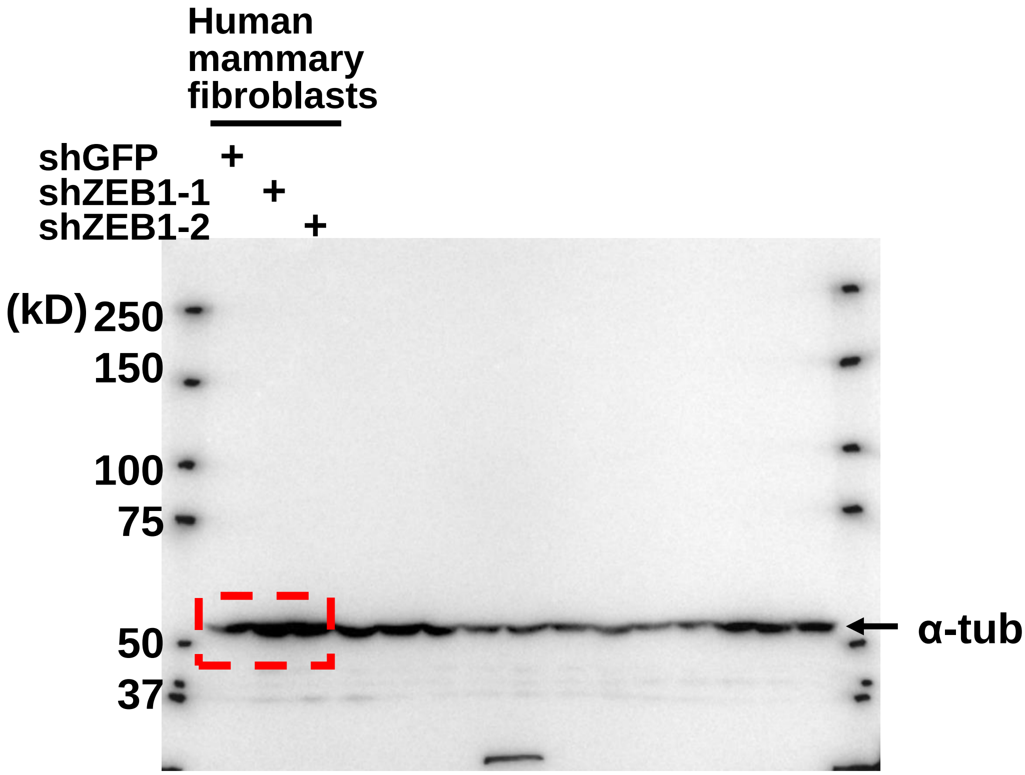

Full unedited gel for Figure 6A

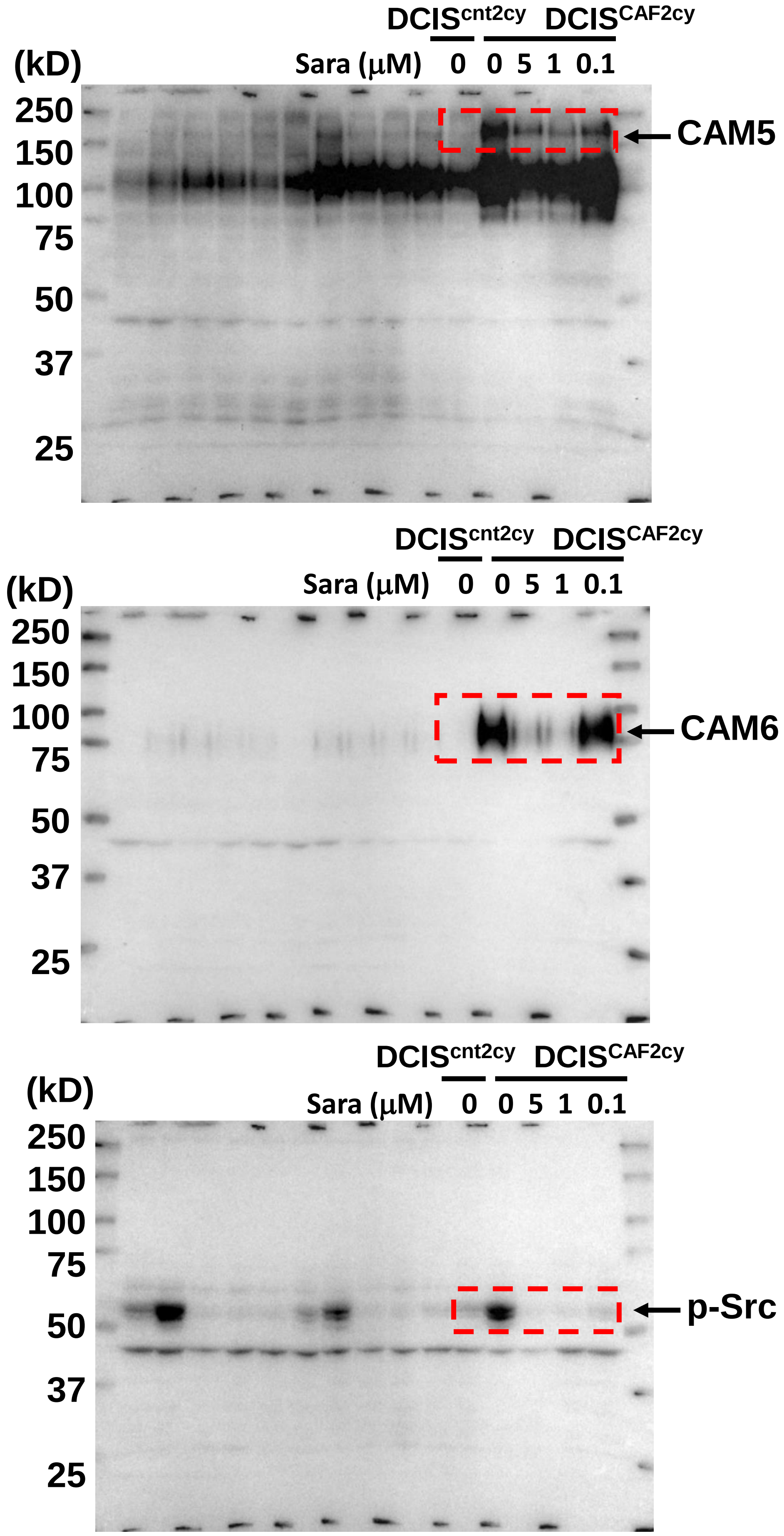

Full unedited gel for Figure 6A

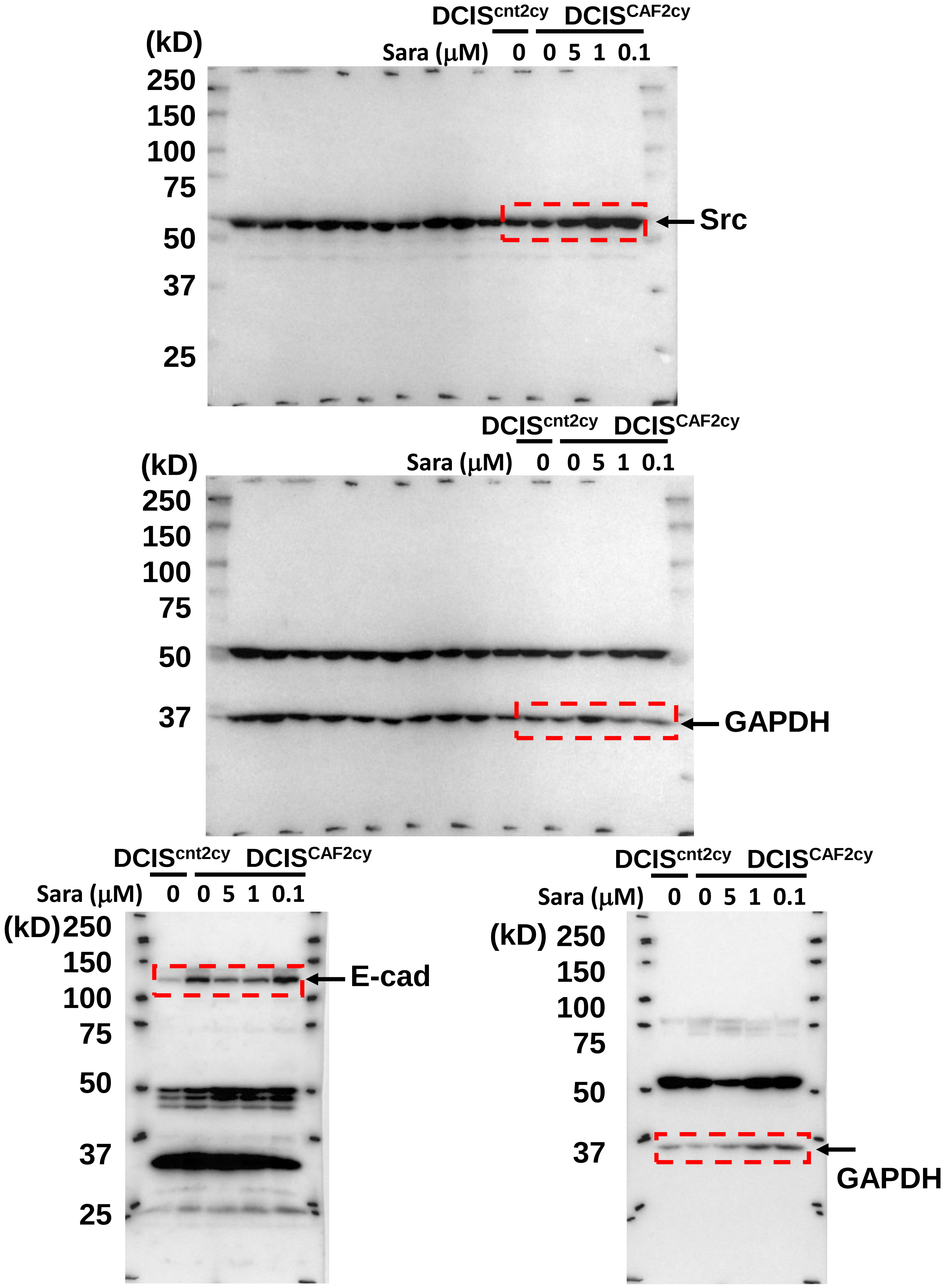

Full unedited gel for Figure 6B

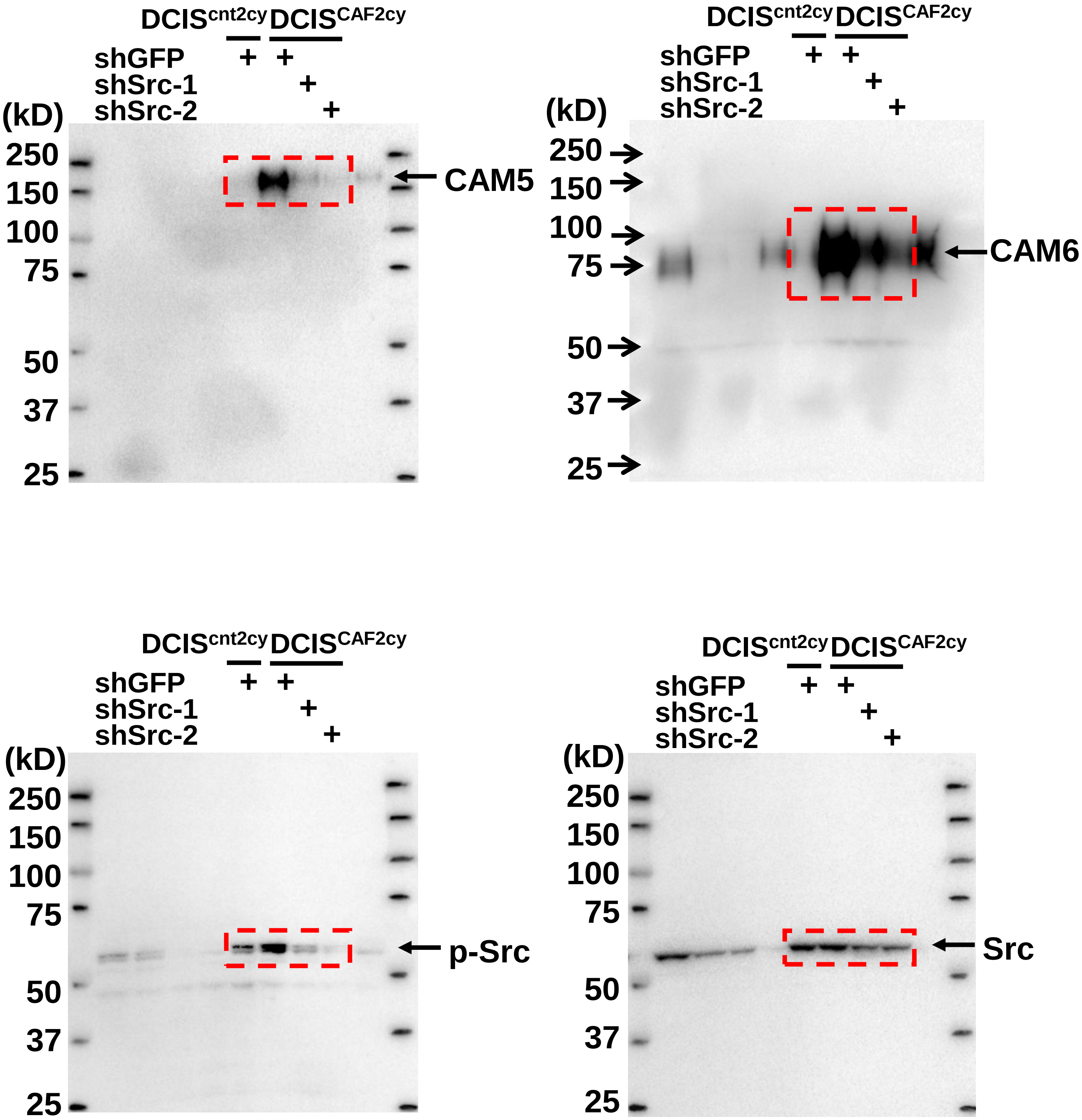

Full unedited gel for Figure 6B

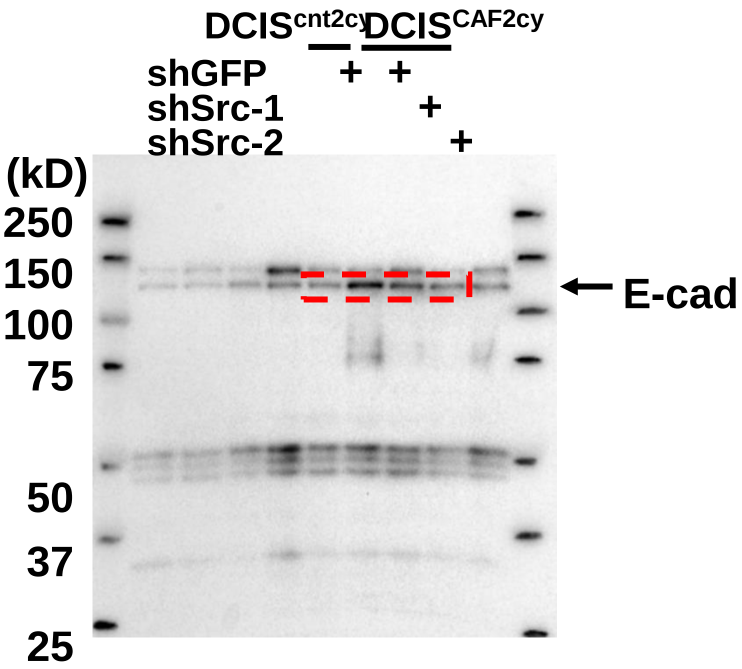

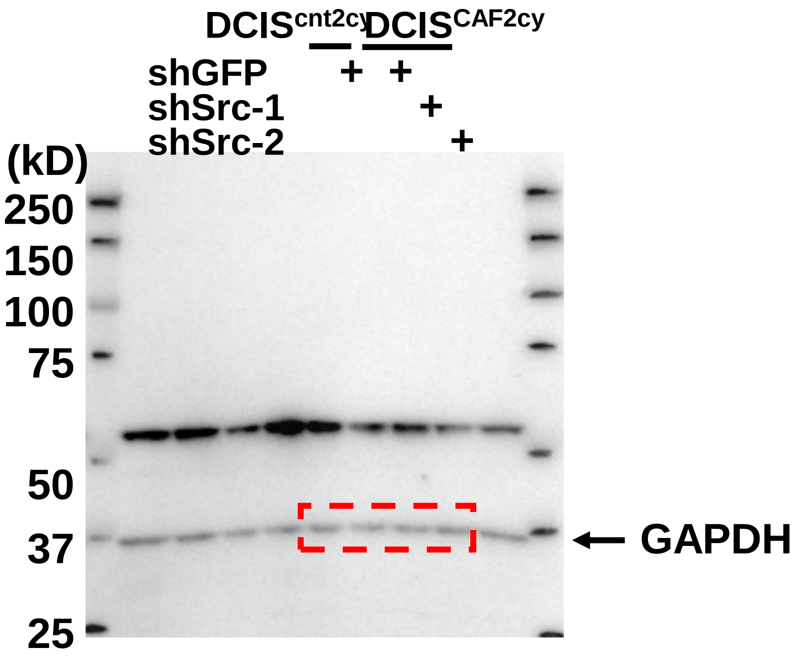

Full unedited gel for Figure 6C (left)

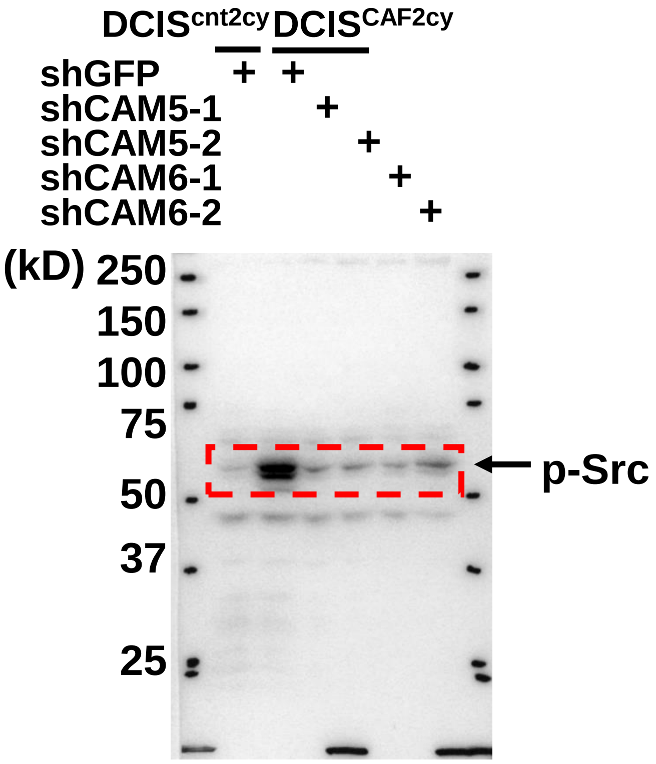

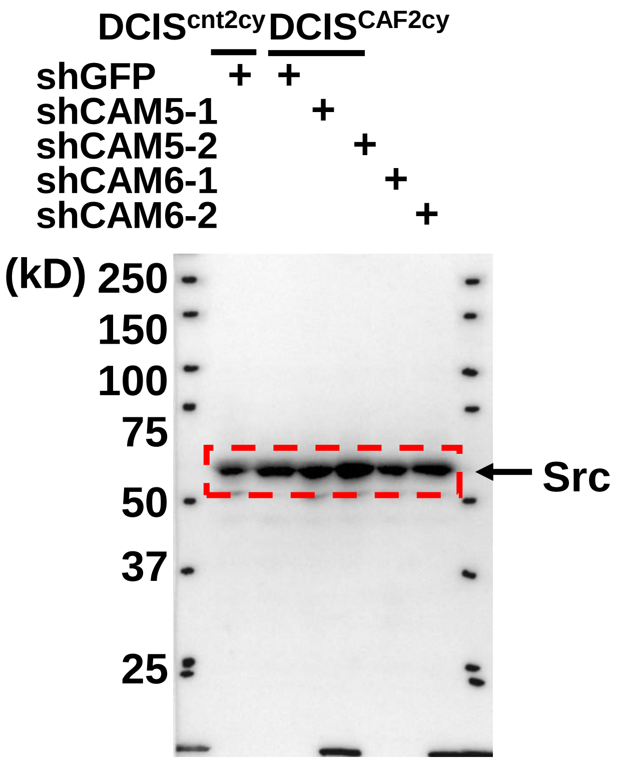

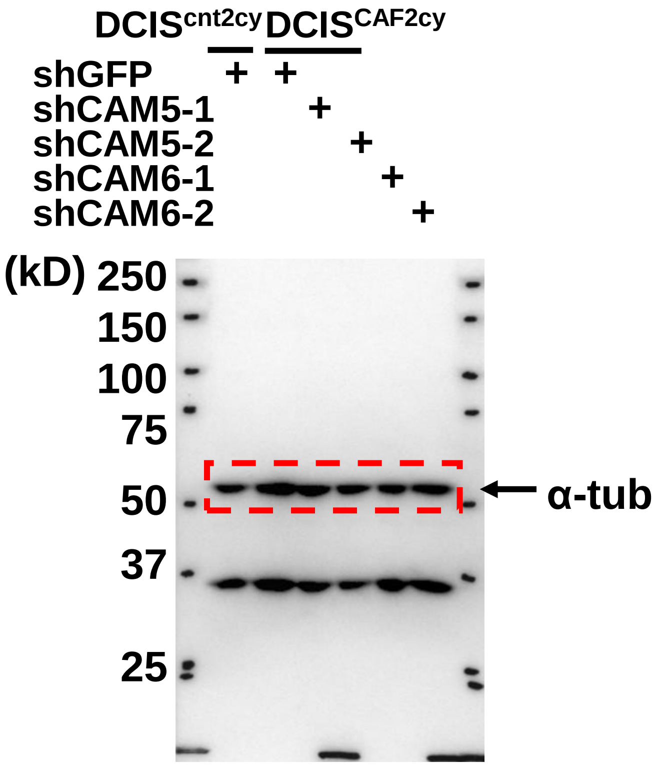

Full unedited gel for Figure 6C (right)

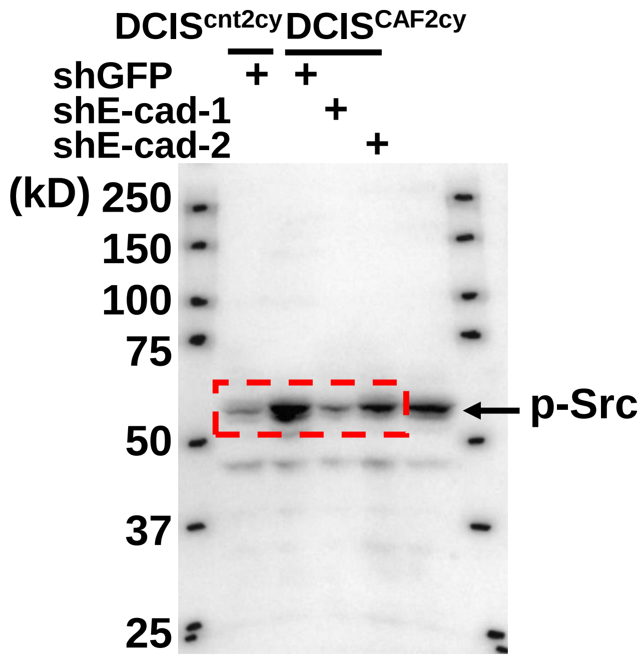

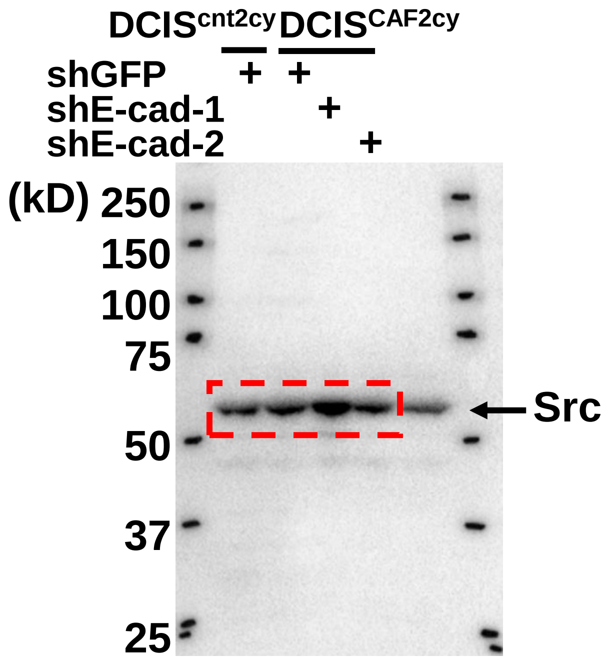

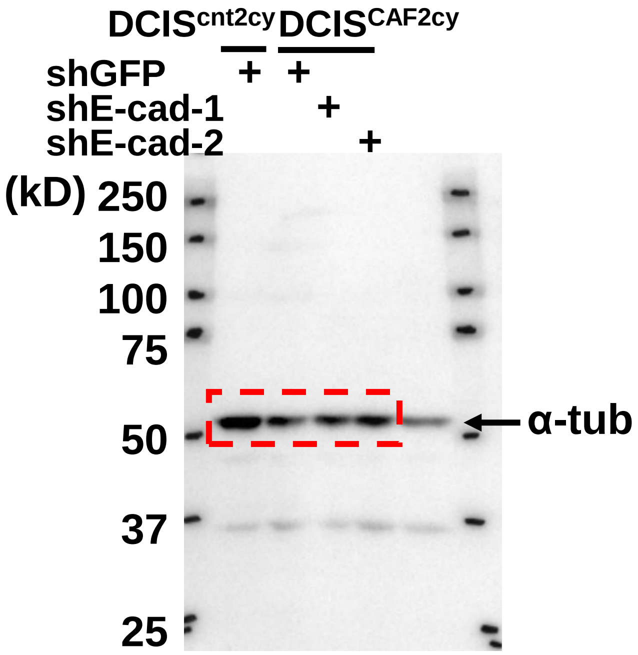

Full unedited gel for Figure 6D

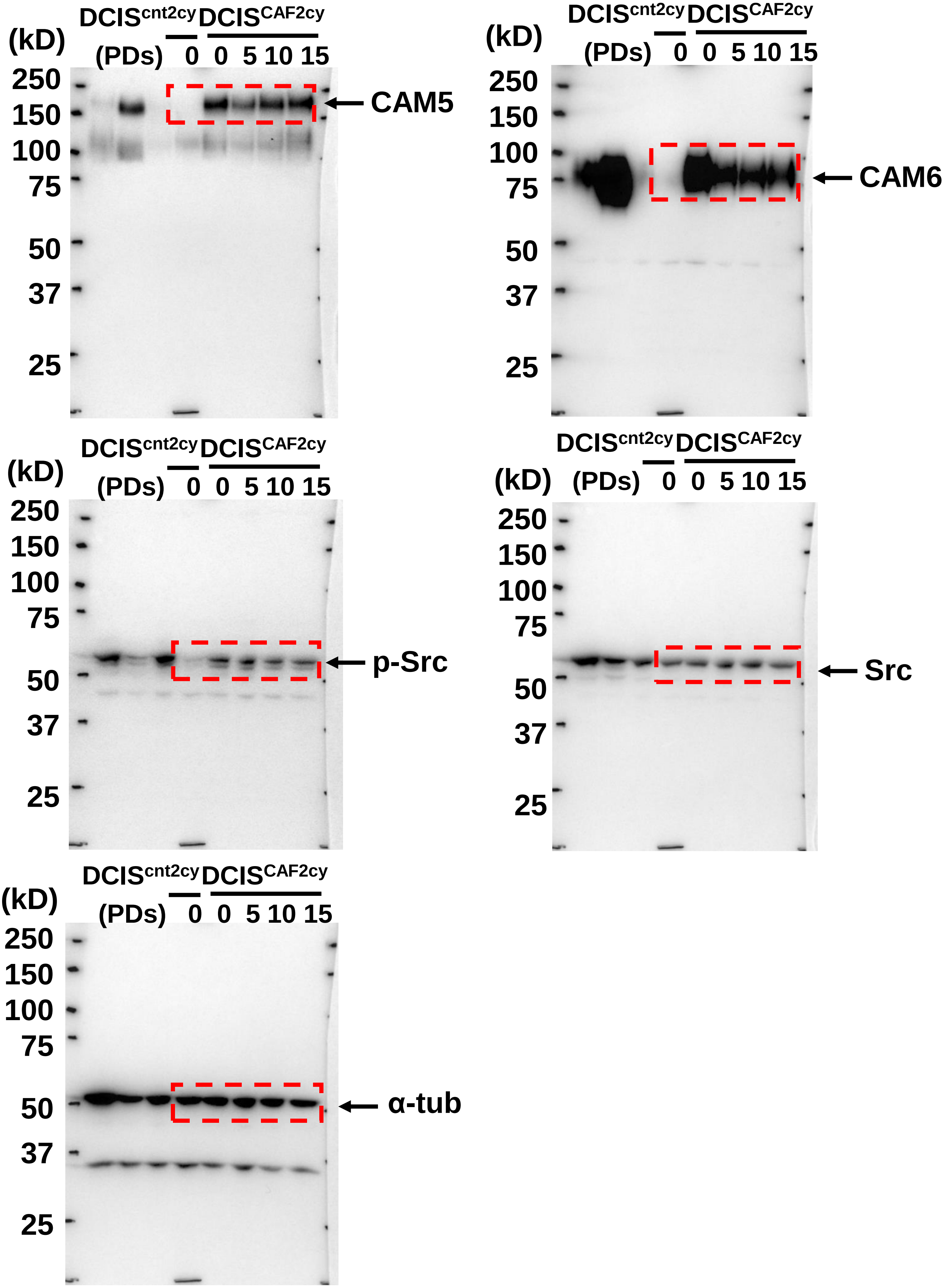

Full unedited gel for Figure 6D

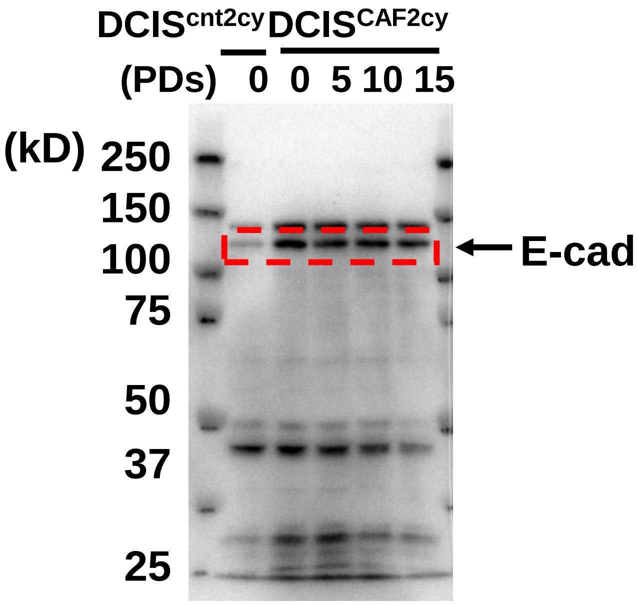

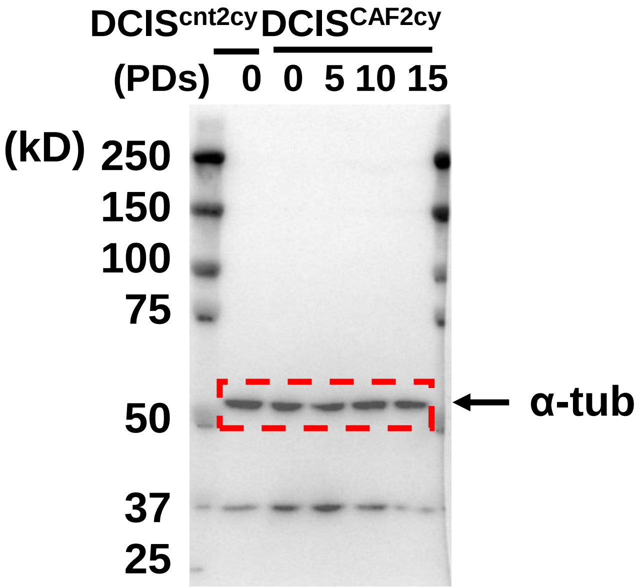

Full unedited gel for Figure 7D

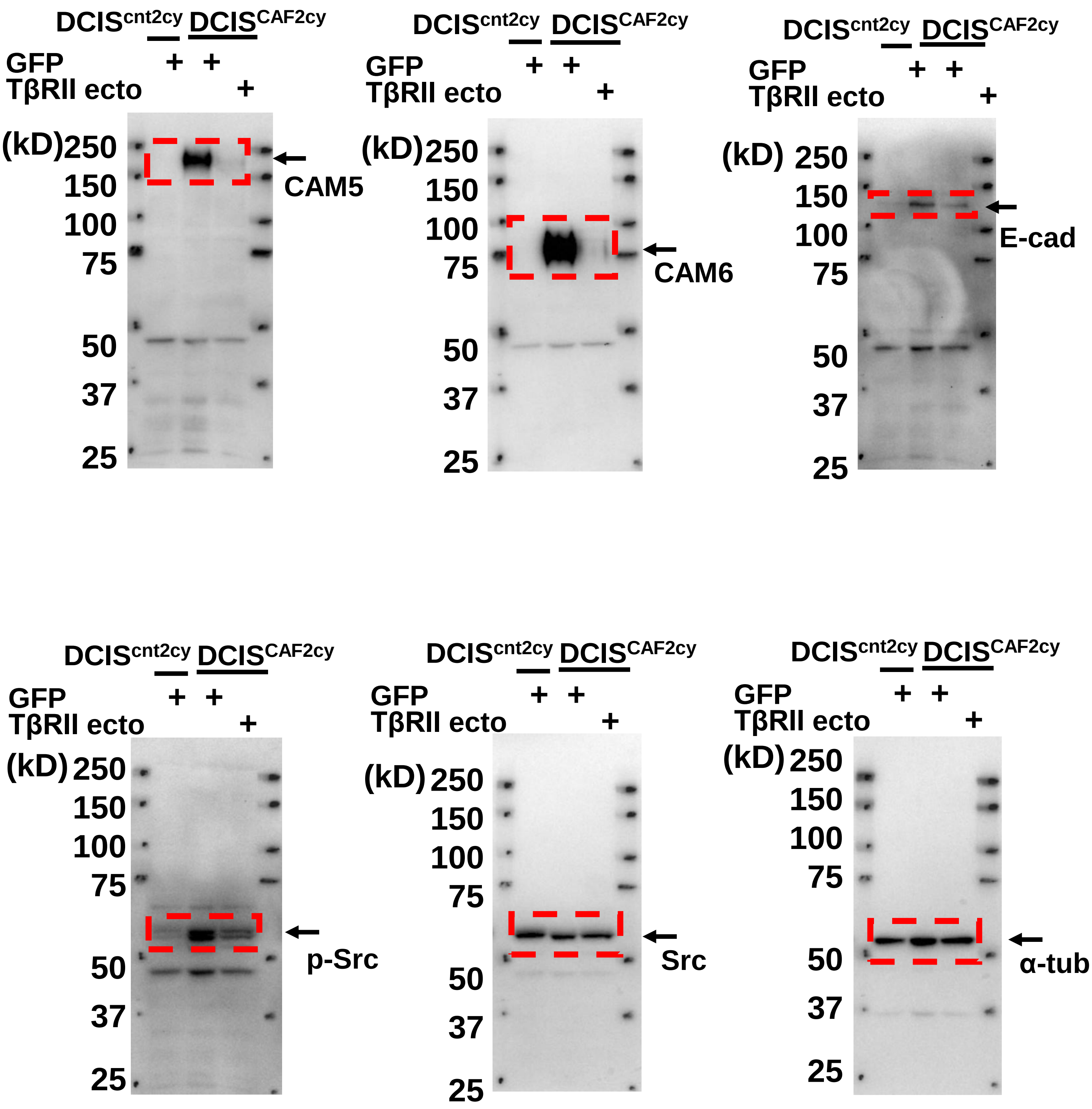

Full unedited gel for Figure 7I

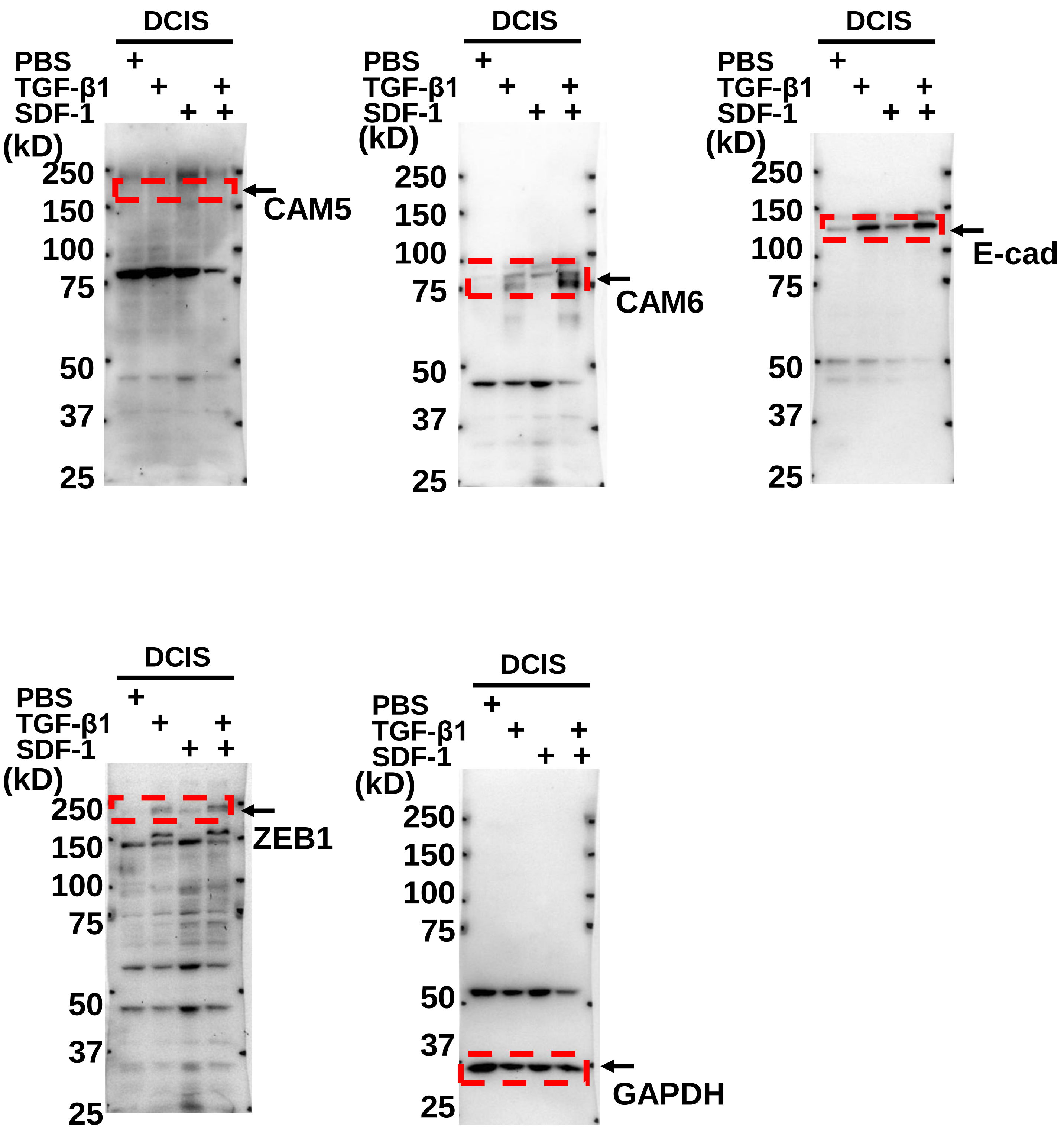

Full unedited gel for Supplemental Figure 5I

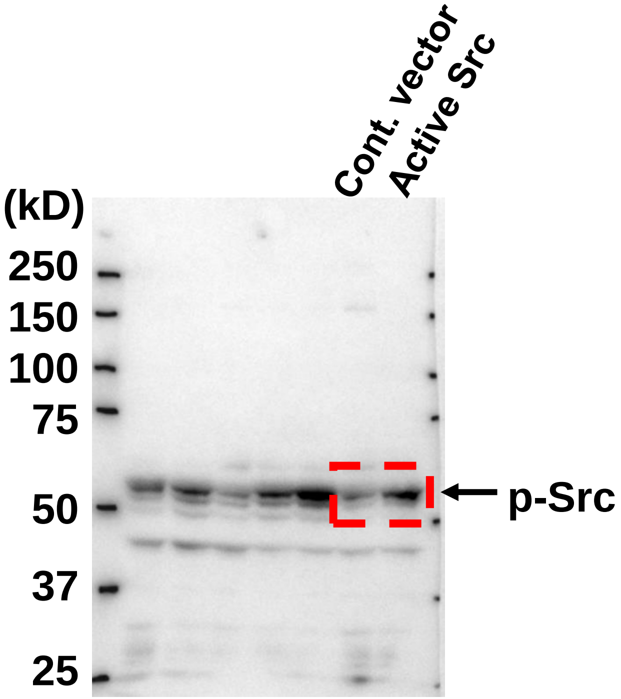

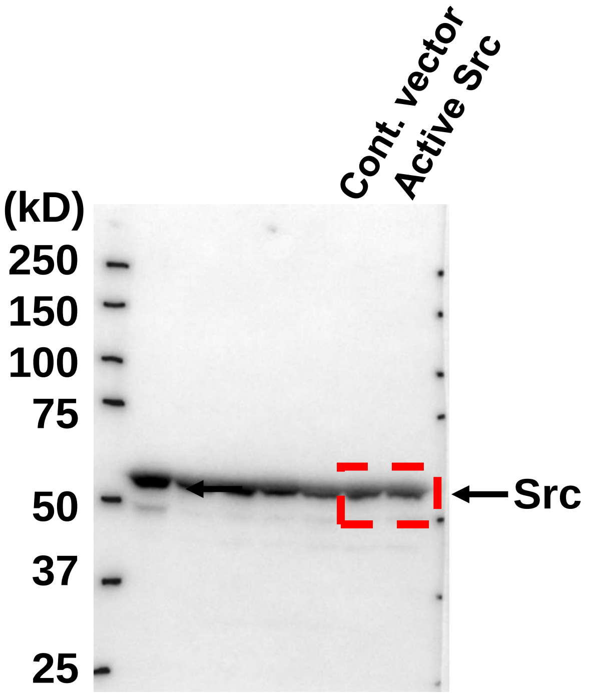

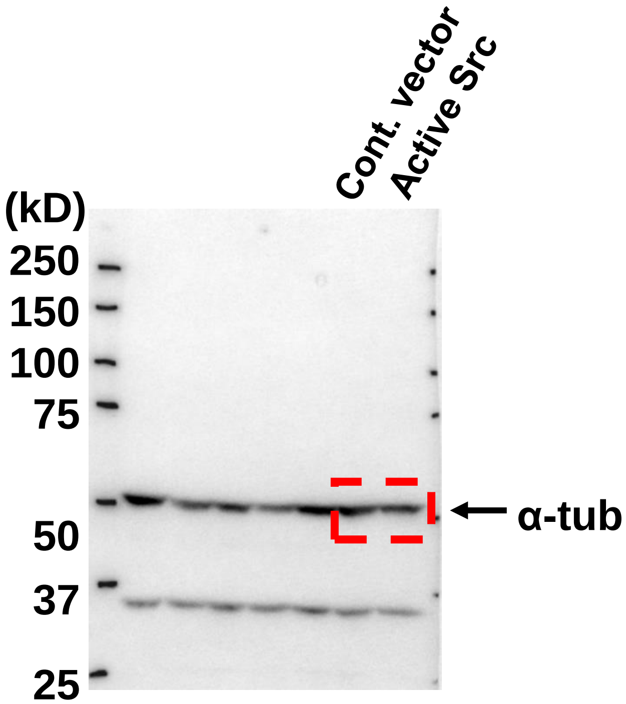

Supplement: Supplementary file 26 [file LSA-2019-00425_SdataF3.pdf]
